# Human intracardiac SSEA4+CD34- cells show features of cycling, immature cardiomyocytes and are distinct from Side Population and C-kit+CD45- cells

**DOI:** 10.1371/journal.pone.0269985

**Published:** 2022-06-16

**Authors:** Mikael Sandstedt, Kristina Vukusic, Benjamin Ulfenborg, Marianne Jonsson, Lillemor Mattsson Hultén, Göran Dellgren, Anders Jeppsson, Jane Synnergren, Joakim Sandstedt

**Affiliations:** 1 Department of Laboratory Medicine, Institute of Biomedicine, Sahlgrenska Academy, University of Gothenburg, Gothenburg, Sweden; 2 Department of Clinical Chemistry, Region Västra Götaland, Sahlgrenska University Hospital, Gothenburg, Sweden; 3 Department of Biology and Bioinformatics, School of Bioscience, University of Skövde, Skövde, Sweden; 4 Department of Molecular and Clinical Medicine, Institute of Medicine, Sahlgrenska Academy, University of Gothenburg, Gothenburg, Sweden; 5 Department of Cardiothoracic Surgery, Region Västra Götaland, Sahlgrenska University Hospital, University of Gothenburg, Gothenburg, Sweden; Massachusetts Eye and Ear Infirmary, Harvard Medical School, UNITED STATES

## Abstract

Cardiomyocyte proliferation has emerged as the main source of new cardiomyocytes in the adult. Progenitor cell populations may on the other hand contribute to the renewal of other cell types, including endothelial and smooth muscle cells. The phenotypes of immature cell populations in the adult human heart have not been extensively explored. We therefore investigated whether SSEA4+CD34- cells might constitute immature cycling cardiomyocytes in the adult failing and non-failing human heart. The phenotypes of Side Population (SP) and C-kit+CD45- progenitor cells were also analyzed. Biopsies from the four heart chambers were obtained from patients with end-stage heart failure as well as organ donors without chronic heart failure. Freshly dissociated cells underwent flow cytometric analysis and sorting. SSEA4+CD34- cells expressed high levels of cardiomyocyte, stem cell and proliferation markers. This pattern resembles that of cycling, immature, cardiomyocytes, which may be important in endogenous cardiac regeneration. SSEA4+CD34- cells isolated from failing hearts tended to express lower levels of cardiomyocyte markers as well as higher levels of stem cell markers. C-kit+CD45- and SP CD45- cells expressed high levels of endothelial and stem cell markers–corresponding to endothelial progenitor cells involved in endothelial renewal.

## 1. Introduction

The human heart is a regenerating organ, as all myocardial cell types–including cardiomyocytes and endothelial cells–are replenished from birth throughout adulthood [1,2]. While the regenerative response greatly diminishes after birth [3], cardiomyocyte renewal holds great potential in treatment of cardiac diseases such as ischemic heart disease and heart failure. As cardiomyocytes are known to be formed mainly from cardiac-resident cell populations, several possible intracardiac sources of physiological cardiomyocyte regeneration have been investigated previously [4]. Proliferation and differentiation of intracardiac C-kit+CD45- [5] and Side Population (SP) [6–8] progenitor cell populations have for example been explored as possible sources for cardiomyocyte renewal. Cardiomyocyte dedifferentiation and proliferation has however subsequently been established as the main mechanism for cardiomyocyte renewal in adult zebrafish [9,10], rodents [11,12] and possibly humans [1].

While cardiomyocyte proliferation is a plausible mechanism for cardiomyocyte renewal in humans, most of the studies have been performed in animal models [9–12]. This is in part due to the absence of a specific marker for cycling human cardiomyocytes as well as the difficulty of performing functional in vivo studies in humans. The phenotype of cycling human cardiomyocytes therefore remains unclear.

Stage-specific embryonic antigens (SSEAs) are markers of immaturity. In a previous study, we described an SSEA4+CD34- cell population in biopsies obtained from right atria in patients undergoing cardiac surgery [13]. These cells were of small cellular size and were characterized by a high gene and protein expression of cardiomyocyte markers. We therefore hypothesize that SSEA4+CD34- cells may constitute a subpopulation of immature cardiomyocytes that might contribute to cardiomyocyte renewal. Our understanding of the phenotype of SSEA4+CD34- cells as well as their relationship to other immature cell populations is still limited. While we demonstrated that the SSEA4+CD34- population was distinct from the C-kit+CD45- population, the relationship to the SP has not yet been investigated. Furthermore, possible differences in SSEA4+CD34- cell phenotype between the four chambers of the human heart, as well as between the healthy and failing heart, have not been explored.

SP cells exist in different human tissues, and may be identified by the capacity to extrude the Hoechst 33342 fluorescent dye through membrane-bound ABC transporter proteins, such as ABCG2 and ABCB1/MDR1 [14–16]. In the adult mouse heart, SP cells have previously been shown to differentiate towards a cardiomyogenic lineage in vitro [7,8] as well as in vivo [6]. An endothelial commitment has also been demonstrated [17,18]. Furthermore, a large portion of studies focused on cardiac progenitor cells have analyzed the C-kit+ cell population. C-kit+ cells were at first shown to be capable of differentiating into cardiomyocytes, endothelial and smooth muscle cells [5]. Later studies, including human studies performed by our group, challenged this concept, implying that cardiac C-kit+ cells are committed to an endothelial lineage and only negligibly contribute to cardiomyocyte renewal [19–23].

It is important to recognize that while cardiac progenitor cell populations may not contribute to formation of new cardiomyocytes, their contribution to other cardiac cell types–including endothelial and smooth muscle cells–may be of importance in healthy as well as in failing hearts. C-kit+CD45- cells as well as SP cells have been investigated mainly in animal models or after in vitro expansion of human cardiac cells. Whether any of these cell populations express markers of stemness and/or lineage differentiation in adult humans is not known. Finally, differences in progenitor cell phenotype between the different heart chambers have not been investigated.

To investigate these questions, we have isolated and analyzed human SSEA4+CD34-, SP and C-kit+CD45- cells from patients undergoing heart transplantation due to heart failure, as well as from donors not suffering from heart failure. This is the first study to extensively characterize the SSEA4+CD34- population as well as to compare multiple human intracardiac progenitor cell populations without prior in vitro expansion.

## 2. Materials and methods

A brief description of the methodology is outlined below, and an overview of the study design is provided in S1 Fig. A more detailed description is provided in the supporting materials and methods in S1 File online.

### 2.1. Tissue procurement

Biopsies from the four heart chambers were obtained from the explanted hearts of patients undergoing heart transplantation at Sahlgrenska University Hospital, after informed written consent. All patients suffered from terminal heart failure (S1 Table). Biopsies were also collected from organ donor hearts not eligible for transplantation, often due to comorbidities or old age. None of the organ donors suffered from chronic heart failure (S2 Table), and all had documentation of consent stating that their organs may be used for other medical purposes than transplantation. The study was approved by the local ethics committee at the University of Gothenburg (#436–15, #596–11) and carried out in accordance with the Helsinki Declaration as revised 2013. Biopsy material from some of the patients were also used in other previously published studies or unpublished studies due to the limited supply of biopsy material [24–26]. No organs/tissues were procured from prisoners.

### 2.2. Cell isolation and staining procedure

The time between excision of the heart and biopsy collection was minimized. Practical aspects including surgical handling and transportation allowed for biopsy collection within a few hours for most hearts, and within 12 hours for all hearts. Excised hearts were stored at 4°C until biopsy collection. Biopsies were collected in cold phosphate buffered saline (PBS), rinsed with PBS in order to remove residual blood, cut into small pieces, digested enzymatically under magnetic stirring and filtered to attain a suspension of single cells. Red blood cells were lysed after dissociation. Cells were filtered using multiple filters down to 40 μm pore size, resulting in an exclusion of larger fragments and cells, including cardiomyocytes. For SP staining, cells were incubated with Hoechst 33342 (Thermo Fisher Scientific, Waltham, MA, USA). In addition, separate negative control samples with Hoechst 33342 and inhibitors of efflux proteins were included. 5 μM Fumitremorgin C (ABCG2 inhibitor, FTC, Sigma-Aldrich St. Louis, MO, USA), 100 μM Verapamil (ABCB1/MDR1 inhibitor, Sigma-Aldrich) and 15 mM sodium azide diluted in 50 mM 2-Deoxy-D-glucose (inhibitor of general metabolism, Sigma-Aldrich) were used as inhibitors of efflux proteins as described previously [27]. Cells which would not undergo staining for SP underwent an epitope regeneration step, in order to optimize detection of protein markers. During antibody staining, cells were incubated with 7-AAD (Thermo Fisher Scientific, for dead cell discrimination) and antibodies (S3 Table).

### 2.3. Flow cytometric analysis and sorting

FACS analysis was carried out on a FACSaria II cell sorter (BD, Franklin Lakes, NJ, USA). Appropriate lasers and filters were used for all fluorochromes included. Data analysis was conducted using FACSdiva version 6.1.1 (BD). An overview of the gating strategy is provided in S2 Fig. Background antibody staining was determined by appropriate isotypic controls. For SP and Main Population (MP), gating was conducted using the most effective inhibitor (verapamil, and sometimes sodium azide/2-Deoxy-D-glucose) to set a morphological SP and MP gate, respectively. Isotypic and inhibitor controls were subtracted when statistics were calculated. After sorting, cell samples were stored in a freezer at –80° C until further analysis.

### 2.4. RNA isolation and gene expression analysis

Kits and equipment from Qiagen (Hilden, Germany) were used to isolate total RNA. Removal of residual genomic DNA was performed using either gDNA columns or DNase1 treatment. Reverse transcription of mRNA to cDNA was performed using the GrandScript cDNA Synthesis Kit (TATAA Biocenter, Gothenburg, Sweden). RNA concentration was measured by digital droplet PCR (Bio-Rad Laboratories, Hercules, CA, USA). All samples were preamplified due to the low concentration of cDNA. Based on a review of the previous literature, 94 gene assays of interest (Thermo Fisher Scientific)–including the reference gene *CYPA*, 11 cardiomyocyte, 11 endothelial and 15 stem cell markers—were selected for analysis (S4 Table). qPCR was performed using the BioMark (Fluidigm, San Francisco, CA, USA) and the 96x96 Dynamic Array™ Integrated Fluidic Circuit at TATAA Biocenter. Cell cycle regulators (S5 Table) were analyzed separately using standard reagents and instruments (Thermo Fisher Scientific).

### 2.5. Statistical analysis

Data were log-transformed prior to further data analysis. For flow cytometric data, groupwise comparisons were carried out using analysis of variance or non-parametric tests. In order to compare the gene expression patterns of different cell populations, as well as to analyze differences between heart chambers and failing/non-failing hearts, unsupervised Principal Component Analysis (PCA), Orthogonal Projections to Latent Structures Discriminant Analysis (OPLS-DA) and hierarchical clustering analysis were used.

Annotations for all genes were manually curated after extensive review of relevant literature. Since genes could have several different annotations, some genes were displayed in several subfigures to visualize annotations. All statistical analyses were performed using Simca v. 15 (Sartorius Stedim Data Analytics AB, Umeå, Sweden) or in R v. 4.0.2 (R Core Team 2020). P values < 0.05 were considered significant.

## 3. Results

### 3.1. SSEA4+CD34-, SP CD45-, SP CD45+ and C-kit+CD45- cells were identified in failing as well as non-failing hearts

**Fig 1:**
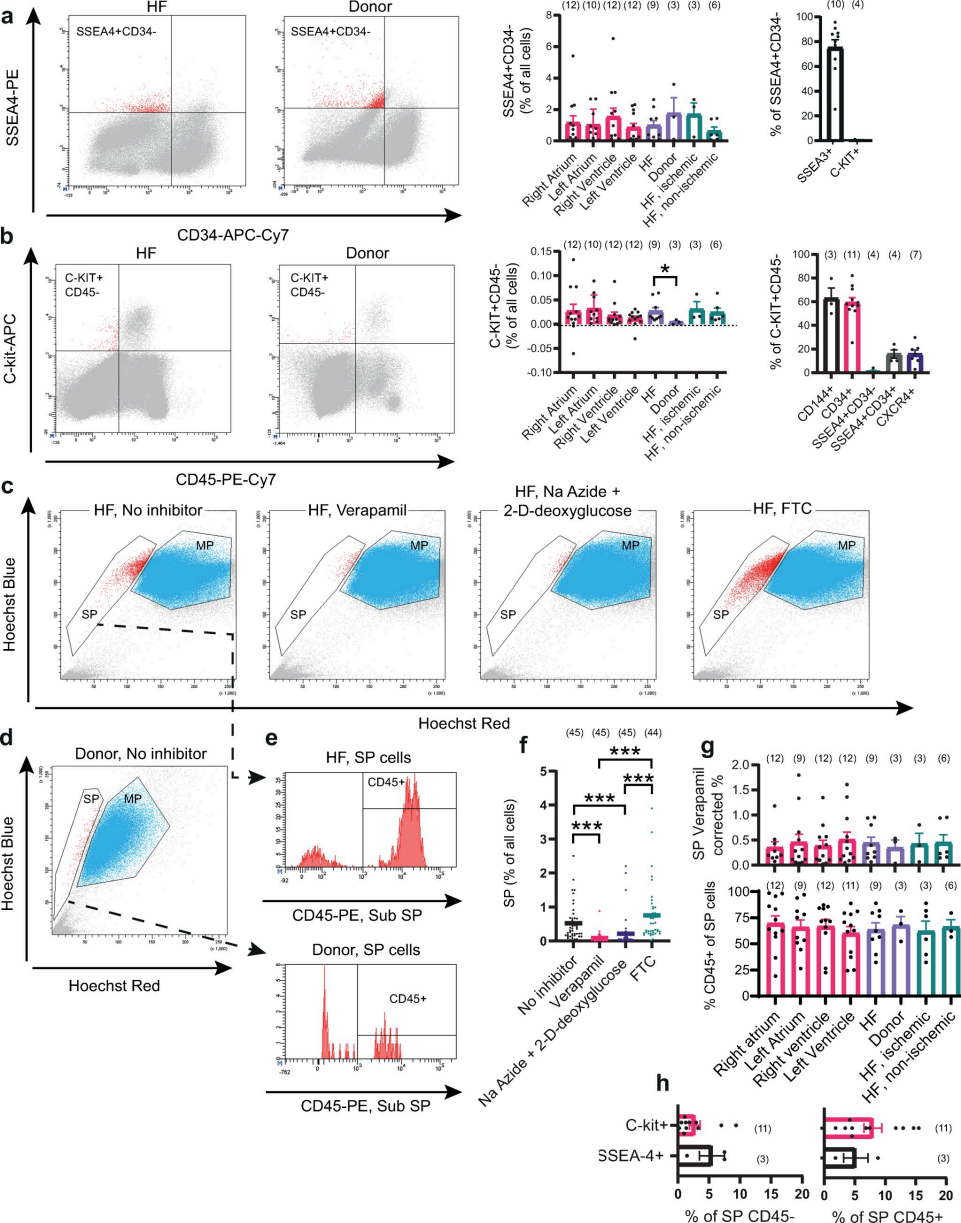
Flow cytometry results. Representative plots (left ventricle) and statistics are shown for all populations that were sorted for subsequent gene expression analysis, as well as some co-stainings. a-b) Results for SSEA4 vs CD34 and C-kit vs CD45 analyses, respectively. To the left, representative FACS-plots. To the right, statistics for all included subjects. c) SP, failing heart. Samples treated with efflux inhibitors are also included. d) SP, donor heart. e) CD45 expression within the SP population. f) Statistics of the SP inhibitor analysis. g) Top: Percentage SP cells corrected for the residual positive cells in Verapamil treated samples. Bottom: Percentage of CD45+ cells of the SP population. h) Percentage of C-kit and SSEA4 positive cells within the SP CD45- and SP CD45+ populations. Statistics are expressed as mean ± standard error of the mean (SEM). Number of study subjects are indicated within parentheses, with the exception of f), where total independent sample numbers are indicated. HF = Heart Failure.

Flow cytometric analysis and sorting of SSEA4+CD34-, SP CD45-, SP CD45+ and C-kit+CD45- cells were conducted. All cell populations were identified in all four chambers of failing as well as non-failing hearts (Figs 1 and S2–S6). C-kit+CD45- cells were significantly enriched in failing hearts, compared to non-failing donor hearts (Fig 1B). The size of the other populations did not differ significantly between failing and non-failing hearts (Fig 1A, 1B and 1G), and there were no significant differences between ischemic and non-ischemic heart failure. C-kit+CD45- cells tended to be enriched in both atria. However, the number of SSEA4+CD34-, SP CD45-, SP CD45+ and C-kit+CD45- cells did not differ significantly between the four chambers of the heart.

The majority of SSEA4+CD34- cells were positive for SSEA3, and the portion of SSEA4+CD34- cells expressing C-kit was negligible (Fig 1A). Endothelial markers CD144/VE-Cadherin and CD34 were expressed by most C-kit+CD45- cells. SSEA4 was only expressed by a small subset of C-kit+CD45- cells, which also expressed CD34 (Fig 1B). The portion of SSEA4+CD34- cells within the C-kit+CD45- cell population was negligible. The chemotactic receptor CXCR4 was also expressed by a smaller fraction of C-kit+CD45- cells. The ABCB1/MDR1 inhibitor verapamil, ABCG2 inhibitor FTC as well as general inhibition of metabolism through sodium azide + 2-D deoxyglucose were used in identifying SP cells (Fig 1C and 1F). Overall, verapamil resulted in the most effective inhibition of Hoechst 33342 efflux, followed by sodium azide + 2-D deoxyglucose. FTC on the other hand, did not result in any significant inhibition of Hoechst 33342 efflux. SP cells in failing as well as non-failing hearts could be further divided into two subpopulations, based on CD45 expression (Fig 1E and 1G). Only a small fraction of SP CD45+ and SP CD45- cells expressed C-kit or SSEA4 (Fig 1H).

### 3.2. SSEA4+CD34-, SP CD45-, SP CD45+ and C-kit+CD45- cells showed distinct gene expression patterns

**Fig 2:**
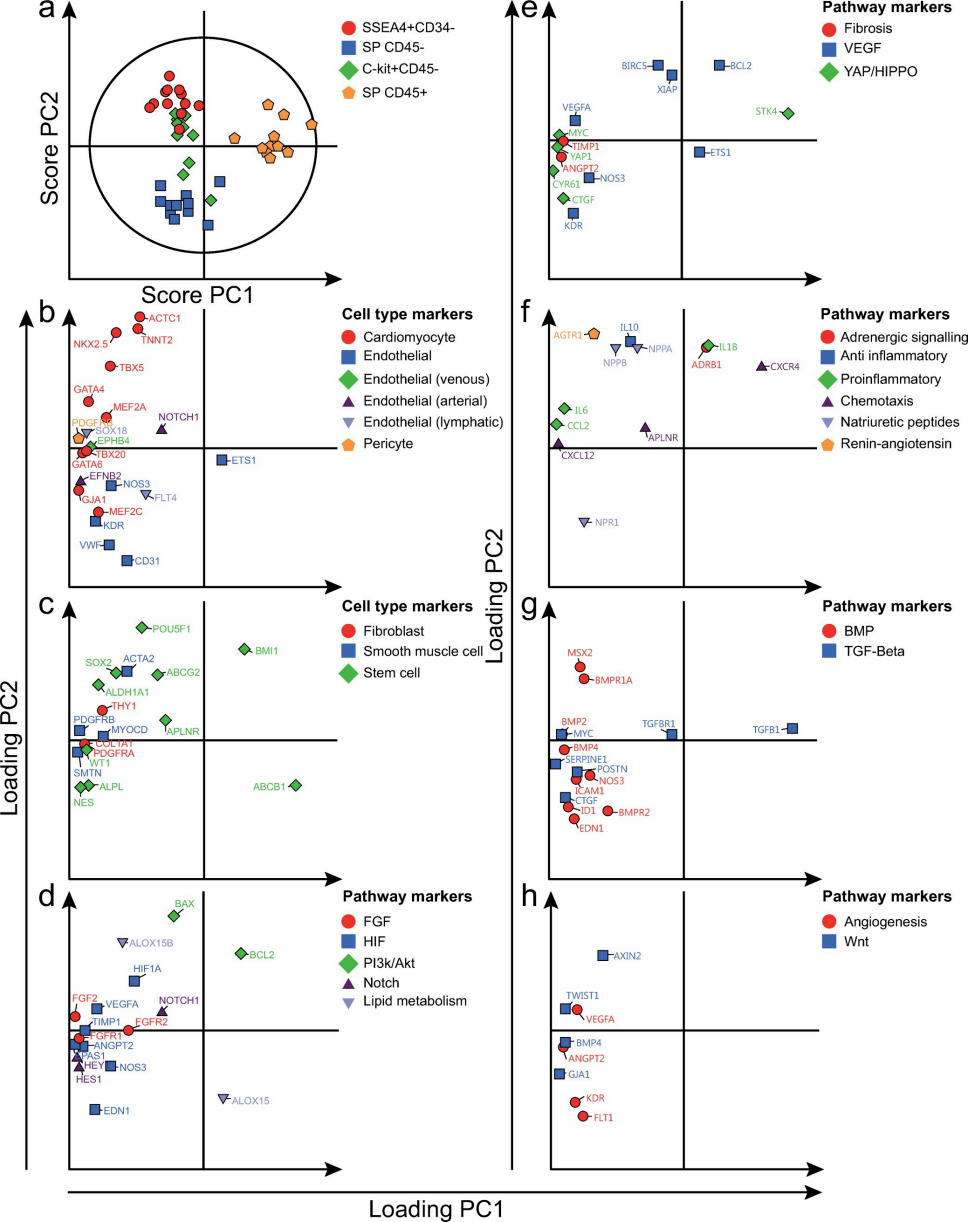
Gene expression patterns of SSEA4+CD34-, SP CD45-, SP CD45+ and C-kit+CD45- cells. Unsupervised PCA model of all four cell populations (included independent cell population datasets = 44). a) Distinct clustering of cell populations, as demonstrated by the two first principal components (PCs) in a score plot. b-h) Gene expression patterns, demonstrated by loading plots. SSEA4+CD34- cells expressed high levels of cardiomyocyte markers. SP CD45- cells on the other hand expressed high levels of endothelial markers. SP CD45+ cells expressed low levels of the majority of analyzed genes, but high levels of certain markers–including *ABCB1*, *CXCR4* and *TGFB1*. C-kit+CD45- cells were not characterized by expression of any specific category of genes. Genes have been color and symbol-coded based on the corresponding gene annotation, as noted to the right of each figure. To improve visualization, some genes are included in more than one panel due to multiple annotations.

**Fig 3:**
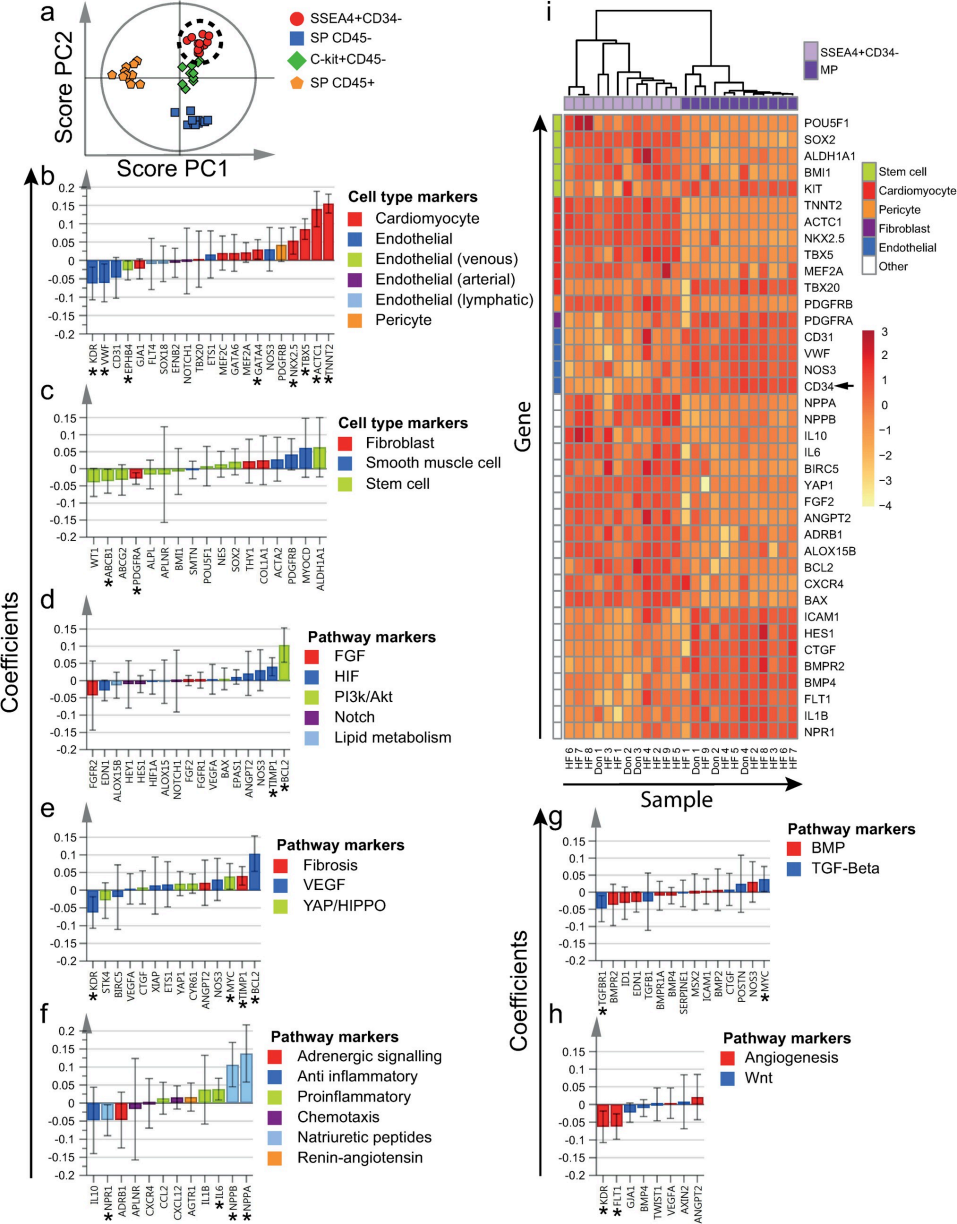
Differentially expressed genes by SSEA4+CD34- cells. a) All four cell populations of interest were included in the supervised OPLS-DA model (included independent cell population datasets = 44). Clustering of SSEA4+CD34- cells, as demonstrated by the two first OPLS-DA predictive components (PCs) in a score plot. b-h) Scaled and centered OPLS-DA regression coefficients with 95% confidence intervals are shown. Significant predicting variables have been marked with "*". i) To determine differentially expressed genes by SSEA4+CD34- cells, MP was used as reference population (included independent cell population datasets = 12). Significantly differentially expressed genes at an FDR of < 5% are included in the heatmap. Hierarchical clustering resulted in separation between SSEA4+CD34- and MP cells. Markers used for flow cytometric sorting are marked with an arrow next to the corresponding row. Genes and populations have been color-coded based on the corresponding annotations, as noted to the right of each figure. To improve visualization, some genes are included in more than one panel, due to multiple annotations. HF = Heart failure patient, Don = Donor.

**Fig 4:**
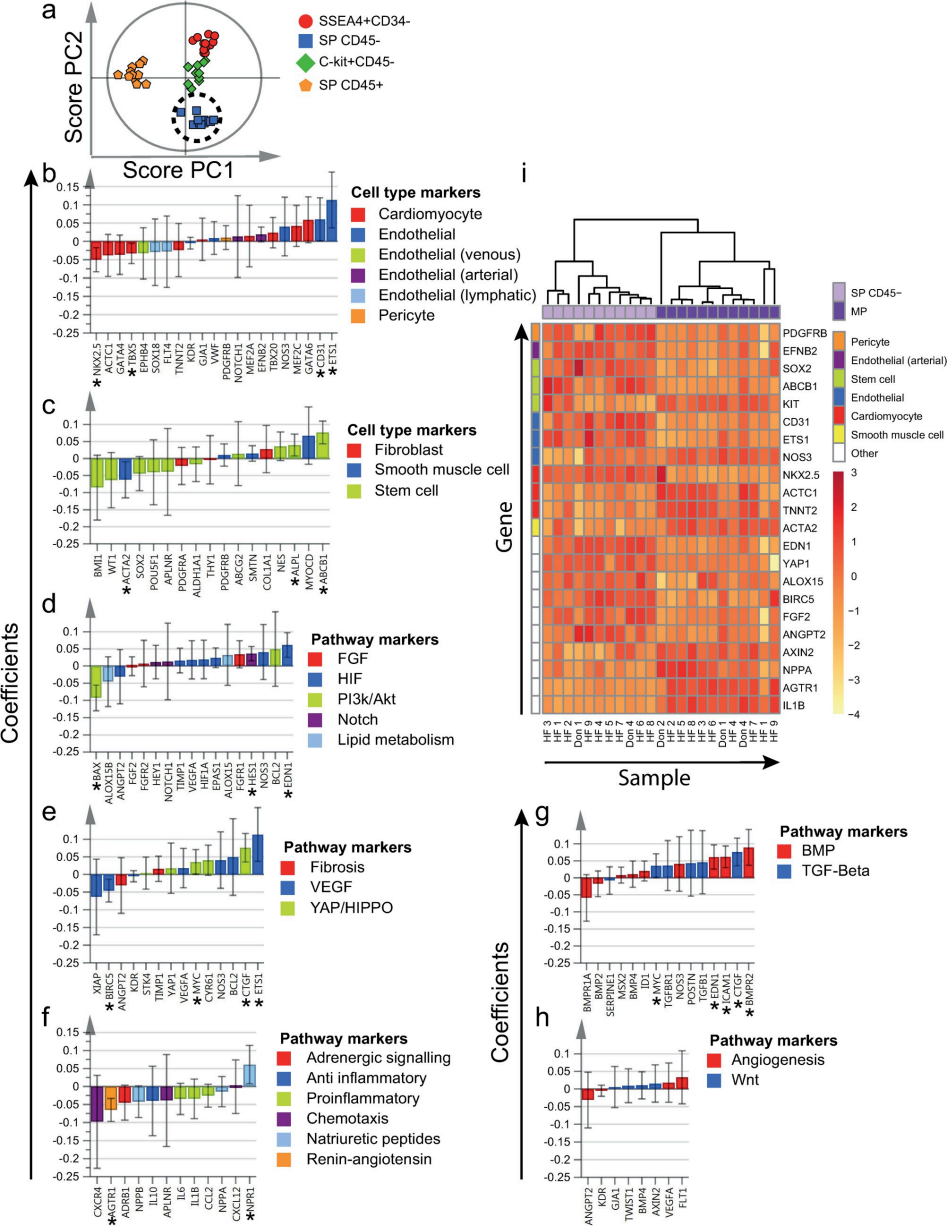
Differentially expressed genes by SP CD45- cells. a) All four cell populations of interest were included in the supervised OPLS-DA model (included independent cell population datasets = 44). Clustering of SP CD45- cells, as demonstrated by the two first OPLS-DA predictive components (PCs) in a score plot. b-h) Scaled and centered OPLS-DA regression coefficients with 95% confidence intervals are shown. Significant predicting variables have been marked with "*". i) To determine differentially expressed genes by SP CD45- cells, MP was used as reference population (included independent cell population datasets = 11). Significantly differentially expressed genes at an FDR of < 5% are included in the heatmap. Hierarchical clustering resulted in separation between SP CD45- cells and MP cells. Genes and populations have been color-coded based on the corresponding annotations, as noted to the right of each figure. To improve visualization, some genes are included in more than one panel, due to multiple annotations. HF = Heart failure patient, Don = Donor.

**Fig 5:**
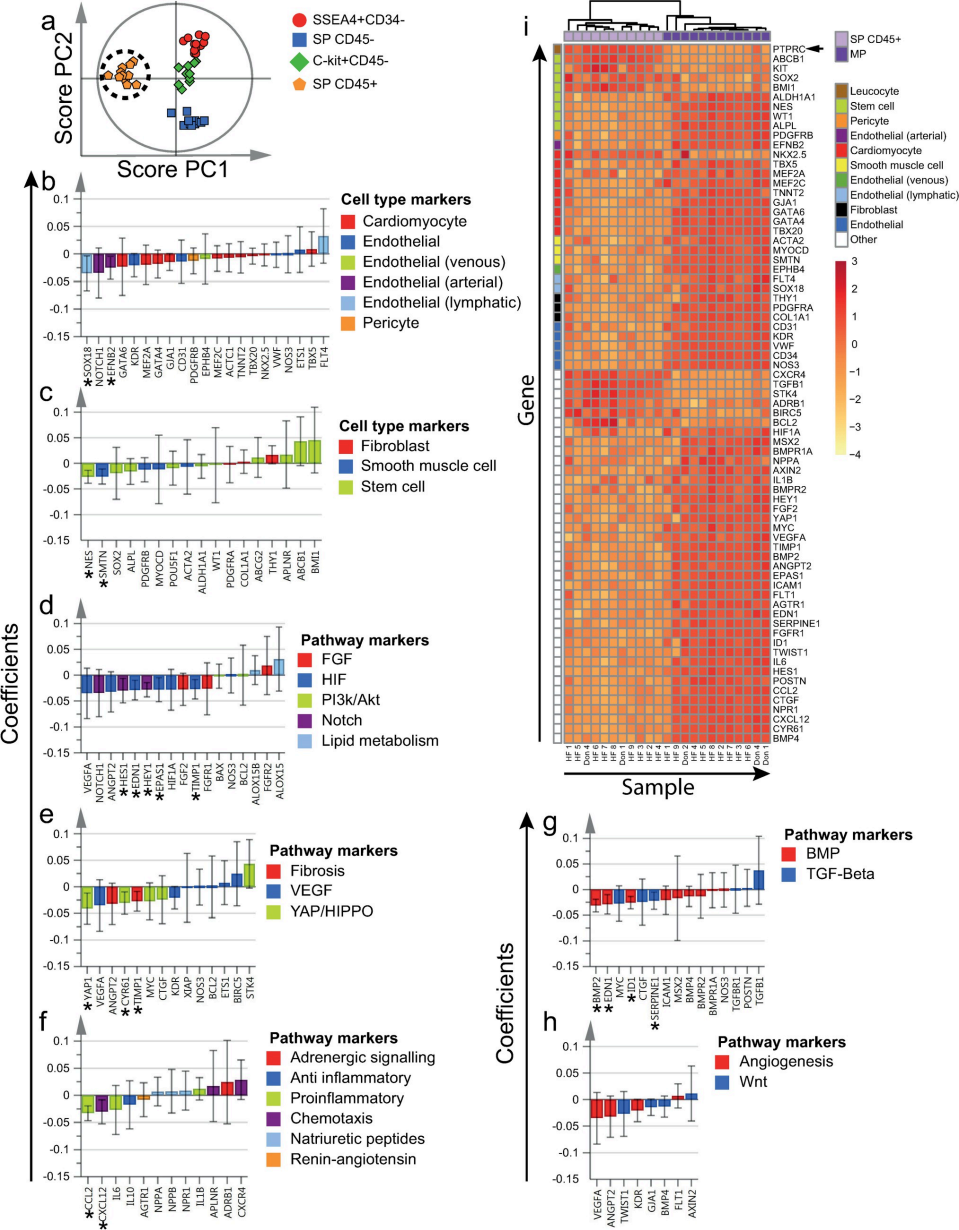
Differentially expressed genes by SP CD45+ cells. a) All four cell populations of interest were included in the supervised OPLS-DA model (included independent cell population datasets = 44). Clustering of SP CD45+ cells, as demonstrated by the two first OPLS-DA predictive components (PCs) in a score plot. b-h) Scaled and centered OPLS-DA regression coefficients with 95% confidence intervals are shown. Significant predicting variables have been marked with "*". i) To determine differentially expressed genes by SP CD45+ cells, MP was used as reference population (included independent cell population datasets = 11). Significantly differentially expressed genes at an FDR of < 5% are included in the heatmap. Hierarchical clustering resulted in separation between SP CD45+ cells and MP cells. Markers used for flow cytometric sorting are marked with an arrow next to the corresponding row. Genes and populations have been color-coded based on the corresponding annotations, as noted to the right of each figure. To improve visualization, some genes are included in more than one panel, due to multiple annotations. HF = Heart failure patient, Don = Donor.

**Fig 6:**
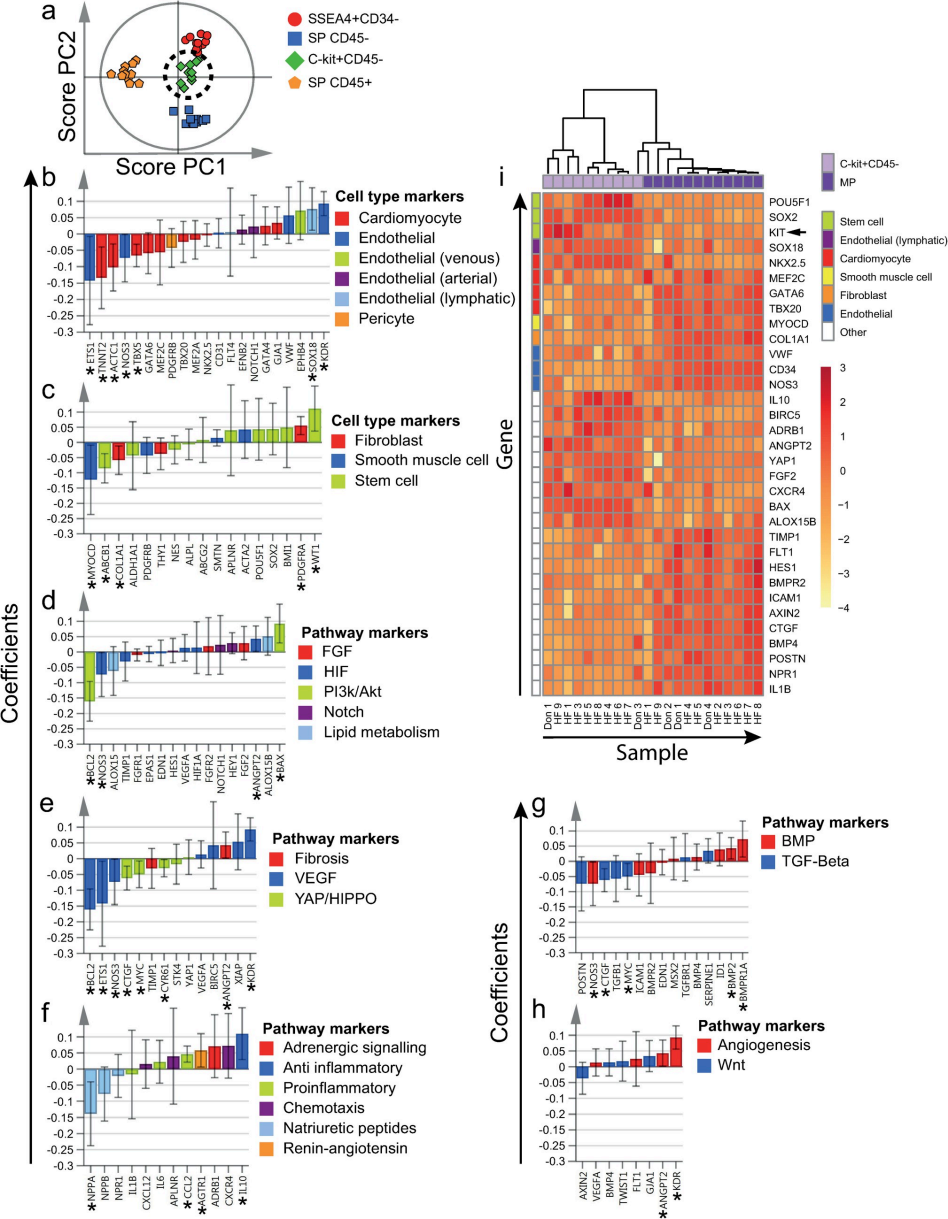
Differentially expressed genes by C-kit+CD45- cells. a) All four cell populations of interest were included in the supervised OPLS-DA model (included independent cell population datasets = 44). Clustering of C-kit+CD45- cells, as demonstrated by the two first OPLS-DA predictive components (PCs) in a score plot. b-h) Scaled and centered OPLS-DA regression coefficients with 95% confidence intervals are shown. Significant predicting variables have been marked with "*". i) To determine differentially expressed genes by C-kit+CD45- cells, MP was used as reference population (included independent cell population datasets = 10). Significantly differentially expressed genes at an FDR of < 5% are included in the heatmap. Hierarchical clustering resulted in separation between C-kit+CD45- cells and MP cells. Markers used for flow cytometric sorting are marked with an arrow next to the corresponding row. Genes and populations have been color-coded based on the corresponding annotations, as noted to the right of each figure. To improve visualization, some genes are included in more than one panel, due to multiple annotations. HF = Heart failure patient, Don = Donor.

An unsupervised PCA model was fitted to assess clustering of cell populations based on gene expression patterns. To further elucidate the expression patterns, a supervised OPLS-DA model was then fitted. The PCA and OPLS-DA models included five and three significant principal/predictive components (PCs), respectively (S7 Fig). SSEA4+CD34-, SP CD45+, SP CD45- and C-kit+CD45- samples formed four distinct clusters based on the two first principal/predictive components (PCA: Fig 2A, OPLS-DA: Figs 3–6A). C-kit+CD45- samples formed a cluster between the three other populations. While the cell populations did not form any distinct clusters based on the third and fourth principal components (S8 Fig), a subset of SP CD45- and SP CD45+ samples tended to cluster together based on the third component.

In order to compare the cell populations with cardiac non-cardiomyocytes, we selected one reference population that included the majority of non-cardiomyocytes. The Main Population (MP) includes the majority of endothelial and hematopoietic cells, as well as fibroblasts, and was therefore used as a reference in all comparisons (Figs 3–6I and S9–12). Hierarchical clustering was performed to illustrate differences between the investigated cell populations and MP.

### 3.3. SSEA4+CD34- cells expressed high levels of cardiomyocyte and stem cell markers

Compared to the other cell populations, SSEA4+CD34- cells expressed higher levels of the majority of cardiomyocyte markers analyzed (Figs 2B and 3B), e.g. *TNNT2*, *ACTC1*, *TBX5*, *NKX2*.*5* and *GATA4*. In contrast, most endothelial markers were expressed at low levels, e.g. *VWF* and *KDR*. Some stem cell markers, e.g. *ALDH1A1*, tended to be highly expressed by SSEA4+CD34- cells, while others tended to be negatively associated, e.g. *ABCB1* (Figs 2C and 3C). Smooth muscle cell markers tended to be expressed at high levels, but regression coefficients were not statistically significant (Figs 2C and 3C). SSEA4+CD34- cells were also characterized by a high expression of *BCL2* (Figs 2D and 3D), proinflammatory cytokines (Figs 2F and 3F), e.g. *IL6*, and the natriuretic peptides *NPPA* and *NPPB*.

Hierarchical clustering resulted in separation of SSEA4+CD34- and MP samples (Figs 3I and S9). The majority of differentially expressed cardiomyocyte markers, *ACTC1*, *TNNT2*, *NKX2*.*5*, *TBX5* and *MEF2A*, were expressed at significantly higher levels in SSEA4+CD34- cells when compared to MP cells (Fig 3I). All differentially expressed endothelial markers, *CD31*, *VWF*, *NOS3* and *CD34*, on the other hand were expressed at lower levels. With the exception of *KIT*, all differentially expressed stem cell markers, *POU5F1*, *SOX2*, *ALDH1A1* and *BMI1*, were expressed at higher levels by SSEA4+CD34- cells. Several pathway markers, including *BCL2*, *BAX*, *YAP1*, *NPPA*, *NPPB*, *IL6*, *IL10*, *CXCR4* and *ADRB1*, were also expressed at higher levels (S9 Fig).

### 3.4. SP CD45- cells expressed high levels of endothelial and stem cell markers

Compared to the other cell populations, SP CD45- cells expressed higher levels of the majority of endothelial markers analyzed (Figs 2B and 4B), e.g. *ETS1* and *CD31*. In contrast, SP CD45- cells expressed several cardiomyocyte markers at low levels, e.g. *NKX2*.*5* and *TBX5*. Several stem cell markers, e.g. *ABCB1* and *ALPL*, were highly expressed by SP CD45- cells (Figs 2C and 4C), while others tended to be lowly expressed. SP CD45- cells also expressed high levels of markers involved in YAP/HIPPO and TGF-β signaling (Figs 2E, 2G, 4E and 4G), e.g. *CTGF* and *MYC*, the natriuretic peptide receptor *NPR1* (Figs 2F and 4F) as well as markers involved in BMP signaling (Figs 2G and 4G), e.g. *BMPR2*, *ICAM1* and *EDN1*.

Hierarchical clustering resulted in separation of SP CD45- and MP samples (Figs 4I and S10). When compared to MP cells, SP CD45- cells expressed higher levels of several endothelial markers, e.g. *CD31*, *ETS1*, *EFNB2*. Two differentially expressed stem cell markers, *SOX2* and *ABCB*1, were also expressed at higher levels. Two cardiomyocyte markers, *ACTC1* and *TNNT2*, were expressed at lower levels in SP CD45- cells, while one, *NKX2*.*5*, was expressed at higher levels. Several pathway markers, including *NPPA*, *AGTR1* and *IL1B* were also expressed at lower levels in SP CD45- cells (S10 Fig). Notably, *YAP1* was expressed at higher levels in SP CD45- cells in comparison to MP cells.

### 3.5. SP CD45+ cells expressed high levels of stem cell markers, but low levels of other cell type specific markers

SP CD45+ cells tended to be negatively associated with the majority of cell type specific markers analyzed (Figs 2B, 2C, 5B and 5C), with the exception of two stem cell markers, *ABCB1* and *BMI1*, which tended to be expressed at high levels. The majority of markers corresponding to pathway annotations also tended to be expressed at low levels (Figs 2D–2H and 5D–5H). Only a few markers, e.g. *STK4*, *CXCR4*, *TGFB1*, tended to be expressed at high levels by SP CD45+ cells.

Hierarchical clustering resulted in separation of SP CD45+ and MP samples (Figs 5I and S11). Hematopoietic cell marker *PTPRC* (CD45) and several stem cell markers, *ABCB1*, *KIT*, *SOX2* and *BMI1*, were expressed at higher levels in SP CD45+ cells when compared to MP cells. Several other differentially expressed stem cell markers as well as the majority of cardiomyocyte, smooth muscle cell, fibroblast and endothelial cell markers, were on the other hand expressed at lower levels. The majority of differentially expressed markers involved in pathways were also expressed at significantly lower levels (S11 Fig). A few markers, including *ADRB1* and *CXCR4*, were however expressed at higher levels by SP CD45+ cells.

### 3.6. C-kit+CD45- cells expressed high levels of stem cell markers, but low levels of cardiomyocyte markers

Compared to the other cell populations, C-kit+CD45- cells expressed lower levels of the majority of cardiomyocyte markers analyzed (Figs 2B and 6B), e.g. *TNNT2*, *ACTC1* and *TBX5*. While the endothelial markers *ETS1* and *NOS3* were expressed at low levels, *VWF* and *KDR* were expressed at high levels. The venous marker *EPHB4* as well as the lymphatic marker *SOX18* were also expressed at high levels. While most stem cell markers were expressed at variable levels (Figs 2C and 6C), *WT1* was expressed at significantly higher and *ABCB1* at significantly lower levels. C-kit+CD45- cells tended to express high levels of *IL10*, *CXCR4*, *CCL2*, *AGTR1*, *ADRB1* and *ANGPT2* (Figs 2F, 2H, 6F and 6H). In contrast, low expression levels were noted for markers involved in YAP/HIPPO (Figs 2E and 6E) and TGF-β (Figs 2G and 6G) signaling, e.g. *CTGF*, *MYC* and *CYR61*, as well as markers involved in natriuretic peptide signaling (Figs 2F and 6F), e.g. *NPPA*.

Hierarchical clustering resulted in separation of C-kit+CD45- and MP samples (Figs 6I and S12). Several stem cell markers, *POU5F1*, *SOX2* and *KIT*, were expressed at higher levels in C-kit+CD45- cells when compared to MP cells. The majority of differentially expressed cardiomyocyte and endothelial markers were on the other hand expressed at lower levels. Notably, the lymphatic endothelial marker *SOX18* was expressed at higher levels in C-kit+CD45- cells. C-kit+CD45- cells also expressed higher levels of *IL10*, *CXCR4* and *ADRB1* (S12 Fig). In contrast, markers involved in TGF-β and BMP signaling were expressed at lower levels.

### 3.7. SSEA4+CD34- cells expressed markers of proliferation and tended to have a more immature phenotype in failing hearts

**Fig 7:**
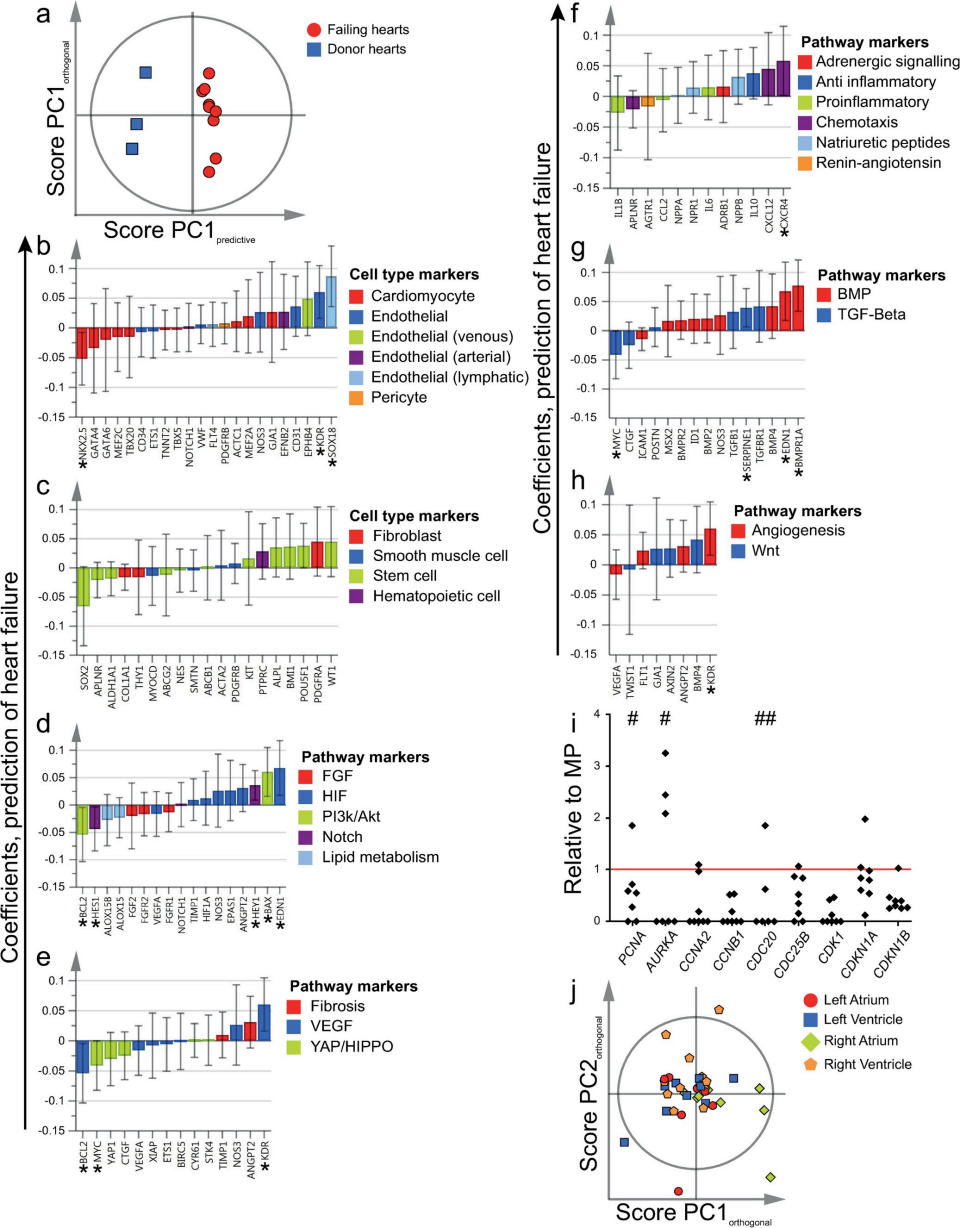
Differences based on heart failure, location and expression of cell cycle regulators. a) SSEA4+CD34- cells from failing and donor hearts were included in an OPLS-DA model (included independent cell population datasets = 12). Clustering of SSEA4+CD34- cells, as demonstrated by the predictive component (PCs) and one orthogonal component in a score plot. b-h) Scaled and centered OPLS-DA regression coefficients with 95% confidence intervals are shown. Significant predicting variables have been marked with "*". i) Cell cycle regulators in SSEA4+CD34- cells from the left atrium and ventricle of failing and non-failing hearts (included independent cell population datasets = 8). Expression level was normalized to the expression level of the corresponding MP (Main Population) sample. The mean expression across all localizations for each heart was plotted. In order to enhance visualization, four data points with very high expression levels were omitted (noted with a “#” for each data point). The red line marks the level of the corresponding MP samples (y = 1). j) SSEA4+CD34- cells isolated from the four different heart chambers were included in an OPLS-DA model (included independent cell population datasets = 36), predicting study participant identity. The orthogonal score plot demonstrated no clustering of samples based on heart chamber identity. Genes and populations have been color-coded based on the corresponding annotations, as noted to the right of each figure. To improve visualization, some genes are included in more than one panel due to multiple annotations.

To analyze whether the phenotype of SSEA4+CD34- cells differed between non-failing and failing hearts, an OPLS-DA model was designed to predict the presence of heart failure based on gene expression. While statistical power was limited as only three donor hearts were included, the resulting model was relatively robust (Figs 7A and S13). In failing hearts, SSEA4+CD34- cells tended to express lower levels of cardiomyocyte markers (Fig 7B), e.g. *NKX2*.*5*, and higher levels of endothelial markers, e.g. *SOX18* and *KDR*. When compared to CD45- and CD45+ SP and C-kit+CD45- cells in failing hearts, SSEA4+CD34- cells were characterized by a high expression of cardiomyocyte markers (S14 Fig). While several stem cell markers such as *WT1*, *POU5F1*, *BMI1* and *ALPL* tended to be expressed at higher levels in failing hearts when compared to non-failing hearts (Fig 7C), others, including *SOX2*, *APLNR* and *ALDH1A1*, were expressed at lower levels. SSEA4+CD34- cells in failing hearts expressed higher levels of markers involved in HIF signaling (Fig 7D), e.g. *EDN1*, *ANGPT2*, *EPAS1* and *NOS3*, as well as in chemotactic signaling (Fig 7F), e.g. *CXCR4* and *CXCL12*. The majority of markers involved in BMP signaling were also expressed at higher levels (Fig 7G). On the other hand, several markers involved in YAP/HIPPO signaling tended to be expressed at lower levels in failing hearts (Fig 7E), e.g. *MYC*, *YAP1* and *CTGF*. SSEA4+CD34- cells expressed cell cycle regulators at varying levels in comparison to the corresponding MP samples (Fig 7I). Notably, while SSEA4+CD34- cells in all analyzed hearts expressed the cell cycle inhibitors *CDKN1A* and *CDKN1B*, SSEA4+CD34- cells in most hearts also expressed the positive cell cycle regulators *PCNA* and *CDC25B*. Several other positive cell cycle regulators were expressed at variable levels. When expression of cell cycle regulators by SSEA4+CD34- cells were compared to that of MP, SP CD45+, SP CD45- and C-kit+CD45- cells through PCA, a model with low reproducibility was obtained (S15 Fig). As no clear segregation of the SSEA4+CD34- cell population from either the MP reference population or the SP CD45- and C-kit+CD45- cell populations was observed, this indicated an overall similar expression pattern of cell cycle regulators. Notably however, SP CD45+ cells tended to cluster separately from the other cell populations based on the first principle component.

### 3.8. Gene expression patterns did not differ between heart chambers, except for the MP and the C-kit+CD45- population

In order to analyze whether the phenotype of SSEA4+CD34-, SP CD45-, SP CD45+ and C-kit+CD45- cells differed between the four chambers of the heart, OPLS-DA models were fitted for each population. The OPLS-DA models were designed to predict study participant identity, in order to correct for the inter-individual variation. As a result, all relevant variation in gene expression explained by the heart chamber identity was included in the orthogonal score and loading vectors, and could therefore be further analyzed.

For SSEA4+CD34- cells (Fig 7J), SP CD45- and SP CD45+ cells (S16 Fig), there was no distinct clustering of samples according to heart chamber identity. C-kit+CD45- cells isolated from the right atrium, however tended to cluster separately from cells isolated from the other chambers of the heart (S16 Fig). This was in part explained by a higher expression of the majority of endothelial markers (S17 Fig), as well as several markers involved in HIF, YAP/HIPPO, chemotactic, BMP and pro-angiogenic signaling. Finally, atrial and ventricular MP cells clustered separately (S16 Fig).

## 4. Discussion

SSEAs are markers of immaturity, expressed at different stages of embryonal tissues and embryonal stem cells (ESCs). SSEA4 and SSEA3 are highly expressed by undifferentiated human embryonic stem cells and are downregulated during differentiation [28]. They are also expressed in human cardiac atrial and ventricular tissue at the fetal and early postnatal stages [29]. We were the first to describe that SSEA4+CD34- cells in the adult human myocardium express high levels of cardiomyocyte markers [13]. In the present study, SP CD45- and SP CD45+ cells did not express SSEA4 to any significant extent. While a small portion of C-kit+CD45- cells expressed SSEA4, these cells also expressed CD34. Based on the flow cytometric and gene expression profile, SSEA4+CD34- cells constituted a clearly-defined population, distinct from C-kit+CD45-, SP CD45+ and SP CD45- cells.

Our results demonstrate a high expression of several cardiomyocyte markers in SSEA4+CD34- cells in comparison to all other progenitor cell populations, as well as to non-cardiomyocyte cells (MP). These markers include transcription factors, mature contractile proteins and natriuretic peptides. In contrast, the expression of endothelial markers was low. The expression pattern of SSEA4+CD34- cells was therefore in line with a cardiomyocyte phenotype. Furthermore, several stem cell markers were expressed at high levels in comparison to the MP. SSEA3 could also be detected based on antibody staining, and SSEA4+CD34- cells expressed cell cycle regulators at similar levels in comparison to the other investigated cell populations. These observations indicate that SSEA4+CD34- cells have a relatively immature phenotype, and that they may have a proliferatory capacity. Previous lineage tracing studies have shown that new cardiomyocytes are formed from cells expressing cardiomyocyte markers such as troponin T, I and myosin heavy chain [12,30,31]. The expression pattern of SSEA4+CD34- cells is in line with results presented in these studies. Based on flow cytometric and gene expression analysis, SSEA4+CD34- cells were evenly distributed between and of similar phenotype in the different chambers of the heart. This pattern is consistent with an immature cardiomyocyte population focused on cardiac regeneration throughout the healthy and failing heart. Interestingly, however, SSEA4+CD34- cells isolated from failing hearts tended to express lower levels of cardiomyocyte markers and higher levels of several endothelial and stem cell markers. Although SSEA4+CD34- cells isolated from failing hearts still expressed cardiomyocyte markers at high levels when compared to the other cell populations, this indicated a less differentiated phenotype in failing hearts. Whether this might be explained by decreased cardiomyocyte maturity or increased restrictions in terminal cardiomyocyte differentiation warrants further study.

Several pathways are considered important for cardiomyocyte reentry into the cell cycle and subsequent proliferation. For example, inhibition of the Hippo pathway has resulted in increased YAP signaling and subsequent stimulation of cardiomyocyte proliferation and survival [32]. This has been shown for example in the setting of myocardial infarction (MI) and in ischemic heart failure in mouse models. The high expression of *YAP1* in SSEA4+CD34- cells was therefore in line with a potential immature, cycling, cardiomyocyte phenotype. SSEA4+CD34- cells isolated from failing hearts tended to express markers involved in YAP/HIPPO signaling at lower levels–perhaps reflecting a lower proliferative potential as compared to non-failing hearts.

Interestingly, several paracrine factors relevant in the pathophysiology of heart failure, including *IL6*, *IL10*, *NPPA* and *NPPB*, were also expressed at higher levels in SSEA4+CD34- cells. These cells may therefore modulate both inflammatory as well as the natriuretic pathways in the failing heart. SSEA4+CD34- cells may also be involved in the SDF-1α/CXCR4 pathway, based on the high expression of *CXCR4*. MI is known to result in elevated SDF-1α/CXCL12 levels, which in turn may result in increased cardiomyocyte survival as well as recruitment of progenitor cells through CXCR4 activation [33]. These mechanisms may also be of importance for the function of SSEA4+CD34- cells in heart failure, based on the upregulation of *CXCL12/CXCR4* in SSEA4+CD34- cells isolated from failing hearts.

While cardiac SP cells have previously been studied in mouse and rat models, few have investigated the human cardiac SP. We have previously described SP cells in the left atrium of patients undergoing Maze procedure due to atrial fibrillation [27]. The specific ABCB1/MDR1 inhibitor verapamil resulted in reduced efflux of Hoechst 33342. ABCB1/MDR1 is also of main importance for the SP phenotype in adult mice, as demonstrated in knockout mice models [16]. In contrast, the ABCG2 inhibitor FTC is known to diminish efflux of Hoechst 33342 for SP cells in the fetal human heart [15]. ABCB1/MDR1 inhibition by verapamil diminished the efflux of Hoechst 33342 in the present study. Furthermore, *ABCB1*, but not *ABCG2*, was upregulated in SP cells, and FTC did not decrease Hoechst 33342 efflux. Taken together, ABCB1/MDR1 seems to be the efflux protein of main importance for the SP phenotype in all four chambers of the failing as well as the non-failing adult human heart.

As the majority of SP cells also expressed the pan-hematopoietic marker CD45, we decided to analyze the SP CD45+ and CD45- subpopulations separately. This has not been the case in several previous studies where the portion of CD45+ SP cells has either been reported to be low [18] or negligible [7,8]. The reason for this discrepancy is unclear, but might be due to differences in species or methodological differences. Our data suggests that subdivision of SP into CD45+ and CD45- is important, as these two subpopulations had distinct gene expression patterns. The hematopoietic phenotype of SP CD45+ cells explains the low expression of the majority of cell type specific markers, whereas the expression of stem markers indicate that the cell population may have a role in the renewal of intracardiac hematopoietic cells. The high expression of the chemokine homing receptor *CXCR4* point toward a migratory capacity of SP CD45+ cells.

In comparison to the other cardiac progenitor populations as well as the MP, SP CD45- cells expressed high levels of endothelial as well as several stem cell markers. An endothelial commitment has indeed previously been established for Hoechst-extruding SP cells in adult mice [17,18]. As we observed that SP CD45- cells were evenly distributed between and of similar phenotype in all heart chambers, SP CD45- cells may have an important role as endothelial progenitor cells in the whole heart. SP cells in adult mice have previously been shown to express immature cardiomyocyte markers and to differentiate towards a cardiomyogenic lineage in vitro [7,8] as well as in vivo [6]. However, several study limitations, including the possibility of artefacts due to impurity at isolation, in vitro expansion, or cell fusion, as well as interspecies differences, have made it difficult to establish a cardiomyogenic potential for human SP cells. In the present study, SP CD45- cells were characterized by a low expression of cardiomyocyte-specific markers. Notably, the MP expressed significantly higher levels of the mature cardiomyocyte markers *TNNT2* and *ACTC1*, while SP CD45- cells expressed significantly higher levels of the immature cardiomyocyte gene *NKX2*.*5*. Although these results do not definitively rule out the possibility for cardiomyocyte differentiation by themselves, SSEA4+CD34- and MP cells demonstrate more cardiomyocyte-like profiles in comparison to SP CD45- cells.

C-kit+CD45- cells expressed low levels of the majority of cardiomyocyte markers analyzed, in comparison to all other cell populations. This is in line with several animal studies that have proposed an endothelial commitment, rather than a cardiomyogenic profile of C-kit+ cells in adult mammals [19–21]. Furthermore, several lineage tracing studies not dependent on stem cell markers have shown that stem-/progenitor cells do not contribute to cardiomyocyte renewal in adult mice to any significant extent [12,30,31,34]. In comparison to SSEA4+CD34- and SP CD45- cells, we found that C-kit+CD45- expressed endothelial and stem cell markers at variable levels. Human C-kit+CD45- cells were more immature as compared to the MP, as shown by the higher expression of several stem cell markers. On the other hand, C-kit+CD45- cells expressed lower levels of several endothelial markers as compared to the MP (which includes endothelial cells) as well as SP CD45- cells. In comparison to SSEA4+CD34- cells however, C-kit+CD45- cells expressed higher levels of endothelial markers. Notably, the C-kit+CD45- cells expressed venous and lymphatic endothelial markers. The endothelial expression pattern of C-kit+CD45- cells was confirmed by flow cytometric analysis, as a majority of C-kit+CD45- cells expressed CD34 and CD144/VE-Cadherin on protein level. Taken together, the C-kit+CD45- cell population emerges as a relatively immature cell population, demonstrating endothelial commitment, albeit not as clear as for SP CD45- cells and mature endothelial cells contained within the MP. Our data suggest that they may have a venous/lymphatic endothelial commitment.

While C-kit+CD45- may serve a role in the whole heart, we observed a tendency of enrichment within the atria. Notably, analysis of orthogonal principal components demonstrated that C-kit+CD45- cells obtained from the right atrium tended to have a more distinct endothelial, pro-angiogenic gene expression profile in comparison to the other heart chambers. Furthermore, the number of C-kit+CD45- cells was also significantly higher in the failing heart–perhaps reflecting an expansion due to angiogenesis and remodeling.

Some limitations with this study should be acknowledged. The access to failing hearts as well as non-failing donor hearts was limited, resulting in a small study size. While study power was limited, the resulting statistical models were overall robust–indicating strong underlying biological differences between the studied cell populations. Modest differences between heart chambers as well as failing and non-failing hearts might however have been too small to observe. Furthermore, we chose to prioritize direct characterization after dissociation, in order to avoid possible contamination and/or in vitro artifacts during in vitro expansion. The limited amount of biopsy material did therefore not allow for any functional in vitro studies. Such future studies need to be conducted to confirm our results. Also, the prolonged experimental protocol, including sample preparation, dissociation, fixation, staining and flow cytometric analysis and sorting could introduce bias in the subsequent gene expression analysis. In a previous study, we however showed that flow cytometric analysis, including fixation, could be combined with subsequent sorting and gene expression analysis of good quality and sensitivity [35]. The impact of the methodological protocol should therefore be limited, particularly as fixation was corrected for during the statistical analysis.

## 5. Conclusions

**Fig 8:**
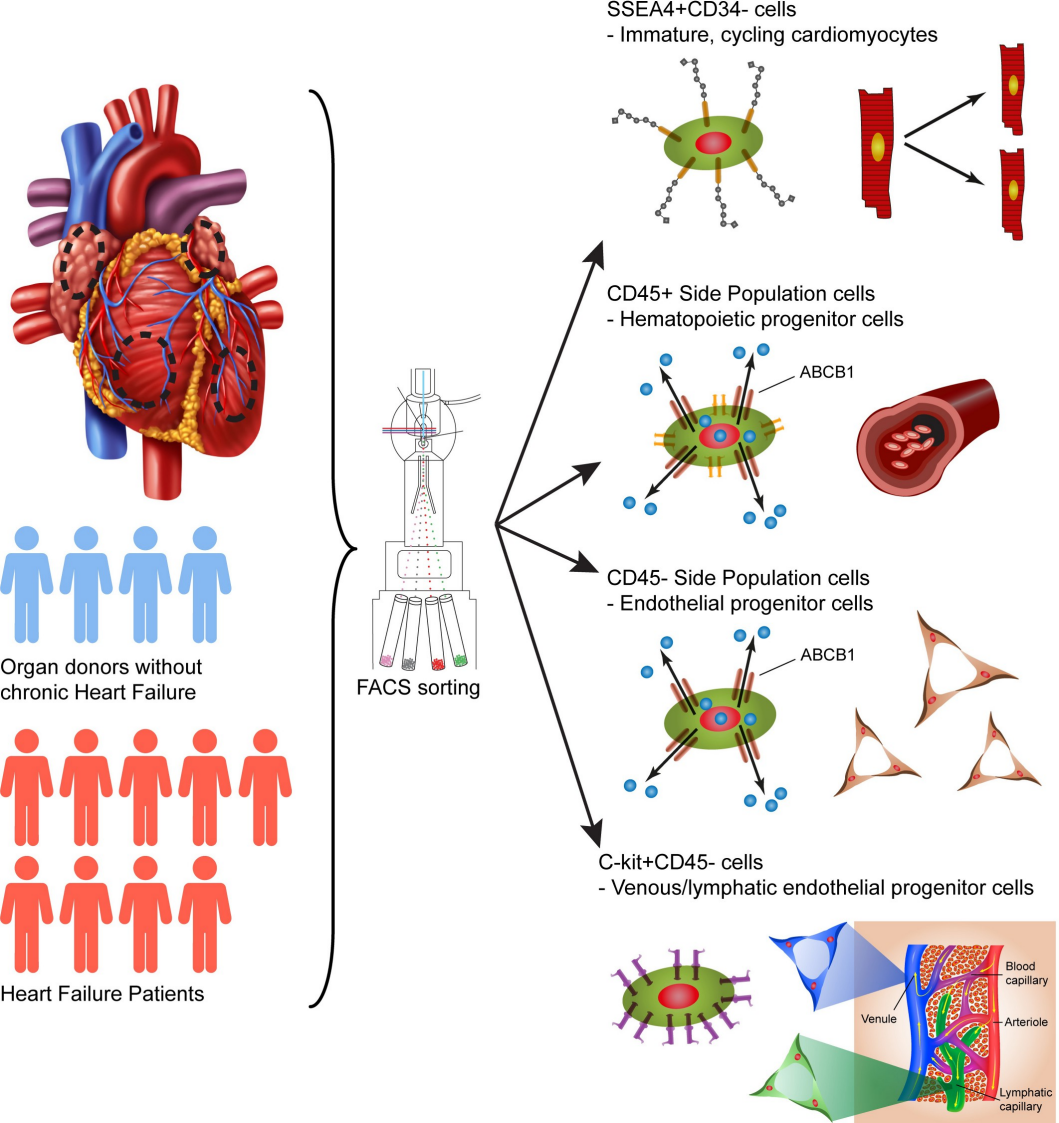
Graphical summary of study findings. SSEA4+CD34-, CD45- and CD45+ SP as well as C-kit+CD45- cells in the four heart chambers of failing and non-failing hearts were characterized through FACS and gene expression analysis. The gene expression pattern of SSEA4+CD34- cells corresponded to that of immature cardiomyocytes, while CD45- SP and C-kit+CD45- cells corresponded to immature endothelial progenitor cells. CD45+ SP cells corresponded to hematopoietic progenitor cells.

In summary, we have demonstrated that SSEA4+CD34-, SP CD45-, SP CD45+ and C-kit+CD45- cells constitute distinct cell populations within the human heart (Fig 8). The gene expression profile of SSEA4+CD34- cells was consistent with that of immature, cycling, cardiomyocytes in all chambers of the heart. SP CD45- as well as C-kit+CD45- cells on the other hand showed features of immature cells with mainly endothelial phenotypes. Finally, we have demonstrated potential signaling pathways that may be important for regulation of the studied populations–including adrenergic, inflammatory, natriuretic peptide, BMP, TGF-β and SDF-1α/CXCR4 signaling.

## Supporting information

S1 Fig Study flow chart. 9 patients undergoing heart transplantation surgery due to heart failure as well as 4 organ donor without chronic heart failure were included as study participants. Biopsies from the available heart chambers were collected for each participant, followed by mechanical and enzymatic dissociation. A portion of the dissociated cells underwent staining for the Side Population (SP) assay, while another portion underwent epitope regeneration followed by staining for cell surface markers. Corresponding control samples were included. Finally, cells underwent FACS analysis and sorting of SSEA4+CD34-, CD45+ and CD45- SP, C-kit+CD45- and Main population (MP) cells. All sorted cell samples underwent qPCR analysis for 94 different genes, which included cell type and pathway markers.
(PDF)

S2 Fig Gating strategy for all cell populations. Illustration of gating strategy by combination of plots from several experiments and multiple cell tubes. Please note that for some experiments, other flourochrome combinations were used in order to enable co-staining with other antibodies. a) Gating strategy of for cell surface markers. DAPI could only be included for fixed, semi-permeable, cells. For non-fixated cells, debris was excluded by FSC vs SSC. b) Gating strategy for SP analysis. Debris was always excluded based on Hoechst staining (gate in lower-left corner, third plot from the left) before calculating the SP percentage of all cells.
(PDF)

S3 Fig SSEA4 and CD34 expression in failing and non-failing hearts. Complete representative set of plots of SSEA4 vs CD34 stainings and corresponding isotypic controls for one failing (a) and one donor heart (b), respectively. Percentages of SSEA4+CD34- cells are noted, without subtraction of isotypic controls. For isotypic controls, percentages are shown for the quadrant corresponding to the SSEA4+CD34- population. Panels to the left constitute isotypic controls. Panels to the right constitute stainings of SSEA4 vs CD34.
(PDF)

S4 Fig C-kit and CD45 expression in failing and non-failing hearts. Complete representative set of plots of C-kit vs CD45 stainings and corresponding isotypic controls for one failing (a) and one donor heart (b), respectively. Percentages of C-kit+CD45- cells are noted, without subtraction of isotypic controls. For isotypic controls, percentages are shown for the quadrant corresponding to the C-kit+CD45- population. Panels to the left constitute isotypic controls. Panels to the right constitute stainings of C-kit vs CD45.
(PDF)

S5 Fig Identification of Side Population cells in the failing heart. Complete representative set of plots of SP stainings including all inhibitors, for one failing heart. Percentage of SP cells (without correction for the residual positive cells in the verapamil treated control sample) is noted for each plot.
(PDF)

S6 Fig Identification of Side Population cells in the non-failing heart. Complete representative set of plots of SP stainings including all inhibitors, for one non-failing donor heart. Percentage of SP cells (without correction for the residual positive cells in the verapamil treated control sample) is noted for each plot. Please note that for this experiment, due to high percentage of debris (incompletely lysed erythrocytes), a live gate was used when collecting data for some of the samples. For these samples, debris in the lower left corner of the plots was excluded. This did not impact sorting or calculation of percentages, as debris in the lower left corner of the SP plots always was excluded by gating strategy (see also S1 Fig) before calculation of SP percentages.
(PDF)

S7 Fig PCA and OPLS-DA model characteristics. All four cell populations were included in an unsupervised PCA model as well as an OPLS-DA model to predict population identity based on gene expression patterns. The PCA model included five significant principal components (a), and the OPLS-DA model included three significant predictive components (b). Cumulative R2X and R2Y were calculated to measure the explained variation of gene expression and population identity, respectively. Cumulative Q2 was calculated to measure the robustness of the models, using cross-validation.
(PDF)

S8 Fig Gene expression patterns of intracardiac SSEA4+CD34-, SP CD45-, SP CD45+ and C-kit+CD45- cells, as visualized by the third and fourth principal components. All four cell populations were included in the unsupervised PCA model (n = 44). a) Cell populations visualized in a score plot of the third and fourth principal components (PC). While there was no distinct clustering of the respective populations, a subset of SP CD45- and SP CD45+ samples tended to cluster together based on the third PC (dashed circle). b-h) Gene expression patterns, demonstrated by loading plots. The cluster of SP CD45- and SP CD45+ samples was associated with high expression of VEGF, BMP and TGF-β markers (dashed circles). Genes have been color-and symbol-coded based on the corresponding gene annotation, as noted to the right of each figure. To improve visualization, some genes are included in more than one panel due to multiple annotations.
(PDF)

S9 Fig Differentially expressed pathway marker genes by SSEA4+CD34- cells. To determine differentially expressed genes by SSEA4+CD34- cells, MP was used as reference population (n = 12). Significantly differentially expressed genes at an FDR of < 5% are included in the heatmap. Several pathway markers, including *BCL2*, *BAX*, *YAP1*, *NPPA*, *NPPB*, *IL6*, *IL10*, *CXCR4*, *ADRB1* were expressed at higher levels in SSEA4+CD34- cells (a-c). The majority of BMP markers were however expressed at lower levels (d). The heat color scale has been centered with a mean of 0 and a standard deviation of 1, for each gene. Hierarchical clustering resulted in separation between SSEA4+CD34- and MP cells. Genes and populations have been color-coded based on the corresponding annotations, as noted to the right of each figure. To improve visualization, some genes are included in more than one panel due to multiple annotations. HF = Heart failure patient, Don = Donor.
(PDF)

S10 Fig Differentially expressed pathway marker genes by SP CD45- cells. To determine differentially expressed genes by SP CD45- cells, MP was used as reference population (n = 11). Significantly differentially expressed genes at an FDR of < 5% are included in the heatmap. Several pathway markers, including *NPPA*, *AGTR1* and *IL1B* were expressed at lower levels in SP CD45- cells (c). *YAP1* on the other hand was expressed at higher levels in SP CD45- cells (b). The heat color scale has been centered with a mean of 0 and a standard deviation of 1, for each gene. Hierarchical clustering resulted in separation between SP CD45- and MP cells in heatmaps for which the number of differentially expressed genes was sufficient for hierarchical clustering (approximately 4–5 genes, a-b). Genes and populations have been color-coded based on the corresponding annotations, as noted to the right of each figure. To improve visualization, some genes are included in more than one panel due to multiple annotations. HF = Heart failure patient, Don = Donor.
(PDF)

S11 Fig Differentially expressed pathway marker genes by SP CD45+ cells. To determine differentially expressed genes by SP CD45+ cells, MP was used as reference population (n = 11). Significantly differentially expressed genes at an FDR of < 5% are included in the heatmap. Most differentially expressed markers were expressed at lower levels in SP CD45+ cells, with a few exceptions–such as *ADRB1* and *CXCR4* (c). The heat color scale has been centered with a mean of 0 and a standard deviation of 1, for each gene. Hierarchical clustering resulted in separation between SP CD45+ and MP cells. Genes and populations have been color-coded based on the corresponding annotations, as noted to the right of each figure. To improve visualization, some genes are included in more than one panel due to multiple annotations. HF = Heart failure patient, Don = Donor.
(PDF)

S12 Fig Differentially expressed pathway marker genes by C-kit+CD45- cells. To determine differentially expressed genes by C-kit+CD45- cells, MP was used as reference population (n = 10). Significantly differentially expressed genes at an FDR of < 5% are included in the heatmap. C-kit+CD45- cells expressed higher levels of *IL10*, *CXCR4* and *ADRB1* (c). Markers involved in TGFβ and BMP signaling were on the other hand expressed at lower levels (d). The heat color scale has been centered with a mean of 0 and a standard deviation of 1, for each gene. Hierarchical clustering resulted in separation between C-kit+CD45- and MP cells. Genes and populations have been color-coded based on the corresponding annotations, as noted to the right of each figure. To improve visualization, some genes are included in more than one panel due to multiple annotations. HF = Heart failure patient, Don = Donor.
(PDF)

S13 Fig OPLS-DA model characteristics. SSEA4+CD34- cells isolated from failing hearts as well as donor hearts were included in an OPLS-DA model to predict presence of heart failure based on gene expression patterns. The model included one significant predictive component and one orthogonal component. Cumulative R2Y was calculated to measure the explained variation of heart failure identity. Cumulative Q2 was calculated to measure the robustness of the model, using cross-validation.
(PDF)

S14 Fig Differentially expressed genes by SSEA4+CD34- cells in failing hearts. a) All four cell populations of interest were included in the supervised OPLS-DA model (included independent cell population datasets = 35). Only samples isolated from failing hearts were included. Clustering of SSEA4+CD34- cells, as demonstrated by the two first OPLS-DA predictive components (PCs) in a score plot. b) Summary of fit, as demonstrated by cumulative R2Y and Q2 for the three included predictive components. c-d) Scaled and centered OPLS-DA regression coefficients with 95% confidence intervals are shown. While pathway markers were included in the OPLS-DA model, only regression coefficients corresponding to cell type markers have been included in the figure. Significant predicting variables have been marked with "*". PC = Predictive Component.
(PDF)

S15 Fig Expression of cell cycle regulators. The expression of 7 positive cell cycle regulators and 2 cell cycle inhibitors were analyzed through qPCR. SSEA4+CD34-, CD45+ and CD45- SP, C-kit+CD45- and MP cell samples from the left atrium and left ventricle of both failing and non-failing hearts were included. A mean delta CT value for each heart was calculated after correction for fixation and presence/absence of heart failure. Prior to PCA model fitting, delta CT values were transformed by adding 1, followed by log transformation, to accommodate transformation of 0 values. Three outliers were excluded based on Hotellings T2 and DmodX. A two component model with low reproducibility was obtained, as demonstrated by the low cumulative Q2 values (a). SSEA4+CD34-, MP, SP CD45- and C-kit+CD45- cells clustered together within the score plot, indicating an overall similar expression pattern of cell cycle regulators (b). SP CD45+ cells tended to cluster separately from the other cell populations. There was no clear correlation between cell population clustering and expression of cell cycle inhibitors or positive cell cycle regulators, as demonstrated by the loading plot (c). PC = Principle Component.
(PDF)

S16 Fig Clustering of samples based on heart chamber identity. SP CD45- (n = 37), SP CD45+ (n = 37), C-kit+CD45- (n = 32) and MP (n = 44) cells isolated from the four different heart chambers were included in four separate OPLSDA models, predicting study participant identity. The orthogonal score plots were used to analyze the clustering of samples based on heart chamber identity. SP CD45- and SP CD45+ samples demonstrated no distinct clustering. C-kit+CD45- cells isolated from right atria tended to cluster separately. MP samples isolated from atria and ventricles clustered separately.
(PDF)

S17 Fig Gene expression patterns of C-kit+CD45- cells based on heart chamber identity. C-kit+CD45- cells isolated from the four different heart chambers were included in an OPLS-DA model (n = 32), predicting study participant identity. a) C-kit+CD45- cells isolated from right atrium tended to cluster separately as shown by the two first orthogonal principal components (PC) in a score plot. b-h) Gene expression patterns, demonstrated by orthogonal loading plots. C-kit+CD45- isolated from right atria expressed high levels of endothelial markers as well as markers involved in HIF, YAP/HIPPO, chemotactic, BMP and pro-angiogenic signaling. Genes have been color- and symbol-coded based on the corresponding gene annotation, as noted to the right of each figure. To improve visualization, some genes are included in more than one panel due to multiple annotations.
(PDF)

S1 Table Clinical background of included heart failure patients. Abbreviations: NYHA, New York Heart Association; LVEF, left ventricular ejection fraction; ACEi, angiotensin-converting enzyme (ACE) inhibitors; ARB, angiotensin receptor blockers; ICD, implantable cardioverter defibrillator; CRT, cardiac resynchronization therapy; CRT-D, cardiac resynchronization therapy defibrillator; PCI, percutaneous coronary intervention; CABG, coronary artery bypass graft surgery.
(PDF)

S2 Table Clinical background of included organ donors. Descriptive statistics for each study participant. No organ donor control suffered from chronic heart failure. Abbreviations: CHF, chronic heart failure; IHD, ischemic heart disease; pMI, previous myocardial infarction; PAD, peripheral artery disease; CVD, Cerebrovascular Disease.
(PDF)

S3 Table Antibodies used for flow cytometric analysis and sorting. Antibodies used for flow cytometric analysis and sorting. Several fluorochrome conjugates were used for different staining panels.
(PDF)

S4 Table Gene assays analyzed using BioMark and the 96x96 Dynamic Array™ IFC. Based on a review of the previous literature, genes of interest (GOI) were selected for analysis. These included markers of cell type as well as pathways relevant for cardiac disease and stem-/progenitor cell biology. 94 assays—including the reference gene *PPIA–*were selected.
(PDF)

S5 Table Cell cycle regulators analyzed using standard qPCR. Based on a review of the previous literature, 9 cell cycle markers were chosen for analysis.
(PDF)

S1 File Supporting material and methods. (DOCX)

S1 Data FACS data. Percentages of FACS analyzed populations.
(XLSX)

S2 Data. 96x96 Dynamic Array data Ct values for the genes analyzed through the 96x96 Dynamic Array™ Integrated Fluidic Circuit. Empty cells constitute undetectable values.
(XLSX)

S3 Data. Cell cycle marker data Ct values for the cell cycle genes analyzed through RT-qPCR. Empty cells constitute undetectable values.
(XLSX)
