# Correction: Human intracardiac SSEA4+CD34- cells show features of cycling, immature cardiomyocytes and are distinct from Side Population and C-kit+CD45- cells

**DOI:** 10.1371/journal.pone.0322513

**Published:** 2025-04-08

**Authors:** The PLOS One Staff

## Notice of Republication

This article was republished on February 21, 2025, to correct error in the title. The publisher apologizes for the error. Please download this article again to view the correct version. The originally published, uncorrected article and the republished, corrected articles are provided here for reference.

## Supporting Information

S1 File Originally published, uncorrected article. (PDF)

S2 File Republished, corrected article. (PDF)

## References

[1] Sandstedt M, Vukusic K, Ulfenborg B, Jonsson M, Mattsson Hultén L, Dellgren G, et al. Human intracardiac SSEA4+CD34- cells show features of cycling, immature cardiomyocytes and are distinct from Side Population and C-kit+CD45- cells. PLoS One. 2022;17(6): e0269985. doi: 10.1371/journal.pone.0269985 35709180 PMC9202910

