# Supplementary material for: Human intracardiac SSEA4+CD34- cells show features of cycling, immature cardiomyocytes and are distinct from Side Population and C-kit+CD45- cells
Source: PLoS One. 2022 Jun 16;17(6):e0269985. doi: 10.1371/journal.pone.0269985 (PMC9202910; doi:10.1371/journal.pone.0269985)
Supplement: S1 Fig — 9 patients undergoing heart transplantation surgery due to heart failure as well as 4 organ donor without chronic heart failure were included as study participants. Biopsies from the available heart chambers were collected for each participant, followed by mechanical and enzymatic dissociation. A portion of the dissociated cells underwent staining for the Side Population (SP) assay, while another portion underwent epitope regeneration followed by staining for cell surface markers. Corresponding control samples were included. Finally, cells underwent FACS analysis and sorting of SSEA4+CD34-, CD45+ and CD45- SP, C-kit+CD45- and Main population (MP) cells. All sorted cell samples underwent qPCR analysis for 94 different genes, which included cell type and pathway markers. (PDF) [file pone.0269985.s001.pdf]

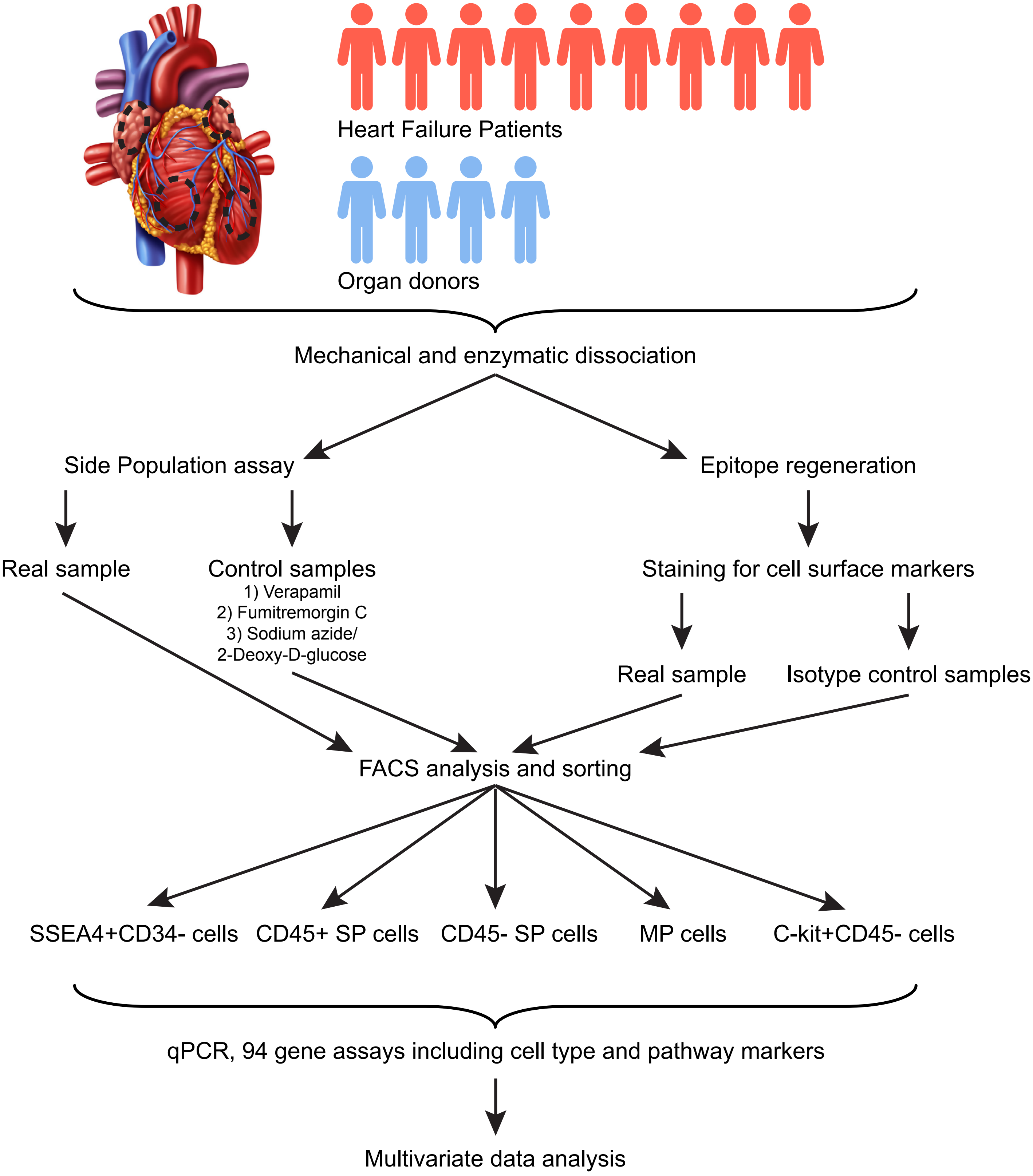
Heart Failure Patients
Organ donors
Mechanical and enzymatic dissociation
Side Population assay
Epitope regeneration
Real sample
Control samples
1) Verapamil
2) Fumitremorgin C
3) Sodium azide/
2-Deoxy-D-glucose
Staining for cell surface markers
Real sample
Isotype control samples
FACS analysis and sorting
SSEA4+CD34- cells
CD45+ SP cells
CD45- SP cells
MP cells
C-kit+CD45- cells
qPCR, 94 gene assays including cell type and pathway markers
Multivariate data analysis

S1 Fig. Study flow chart.

9 patients undergoing heart transplantation surgery due to heart failure as well as 4 organ donor without chronic heart failure were included as study participants. Biopsies from the available heart chambers were collected for each participant, followed by mechanical and enzymatic dissociation. A portion of the dissociated cells underwent staining for the Side Population (SP) assay, while another portion underwent epitope regeneration followed by staining for cell surface markers. Corresponding control samples were included. Finally, cells underwent FACS analysis and sorting of SSEA4+CD34-, CD45+ and CD45- SP, C-kit+CD45- and Main population (MP) cells. All sorted cell samples underwent qPCR analysis for 94 different genes, which included cell type and pathway markers.
