# Supplementary material for: Human intracardiac SSEA4+CD34- cells show features of cycling, immature cardiomyocytes and are distinct from Side Population and C-kit+CD45- cells
Source: PLoS One. 2022 Jun 16;17(6):e0269985. doi: 10.1371/journal.pone.0269985 (PMC9202910; doi:10.1371/journal.pone.0269985)
Supplement: S2 Fig — Illustration of gating strategy by combination of plots from several experiments and multiple cell tubes. Please note that for some experiments, other flourochrome combinations were used in order to enable co-staining with other antibodies. a) Gating strategy of for cell surface markers. DAPI could only be included for fixed, semi-permeable, cells. For non-fixated cells, debris was excluded by FSC vs SSC. b) Gating strategy for SP analysis. Debris was always excluded based on Hoechst staining (gate in lower-left corner, third plot from the left) before calculating the SP percentage of all cells. (PDF) [file pone.0269985.s002.pdf]

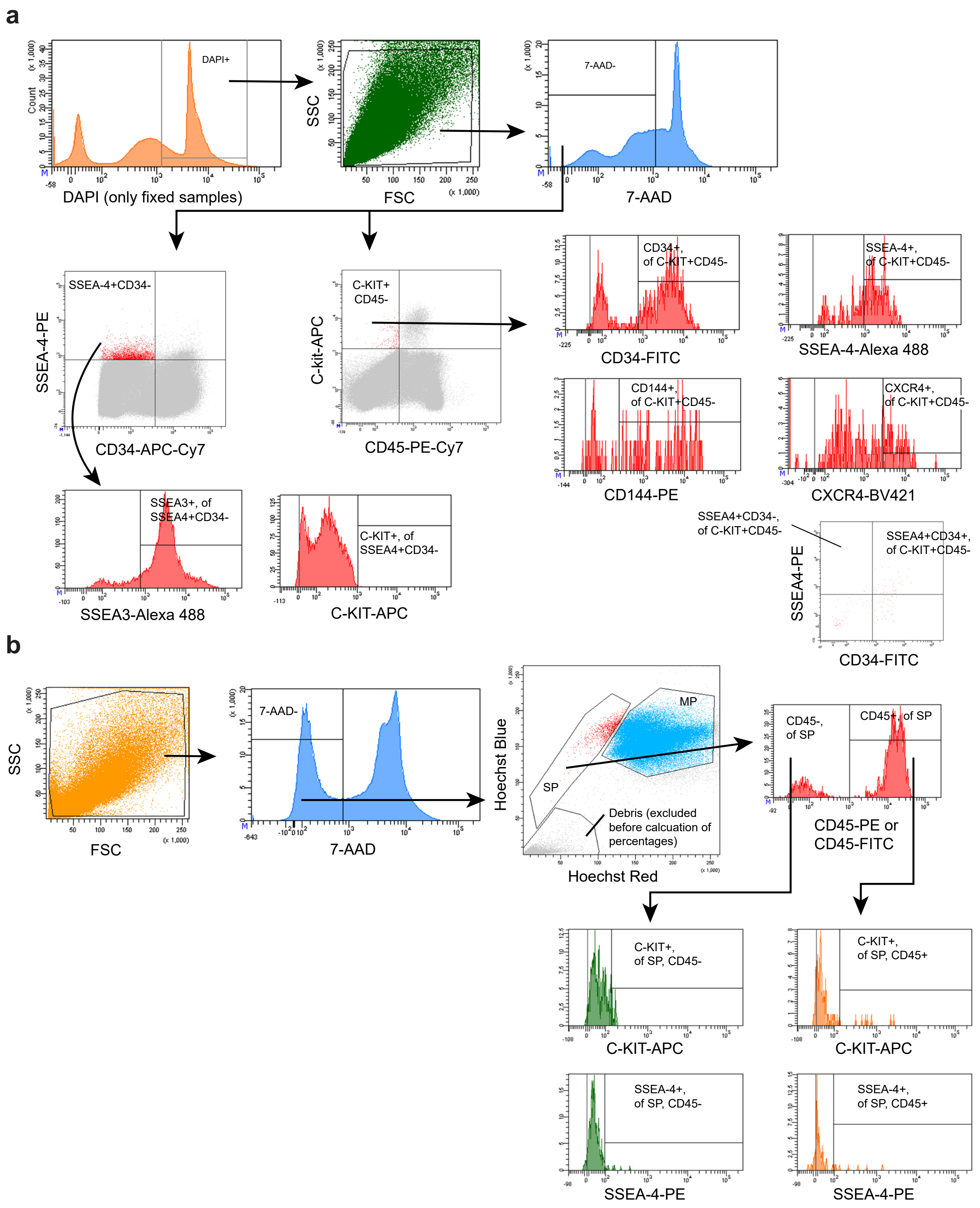

a
DAPI+
Count
DAPI (only fixed samples)
SSC
FSC
7-AAD-
7-AAD
SSEA-4+CD34-
SSEA-4-PE
CD34-APC-Cy7
C-KIT+
CD45-
C-kit-APC
CD45-PE-Cy7
CD34+,
of C-KIT+CD45-
CD34-FITC
SSEA-4+,
of C-KIT+CD45-
SSEA-4-Alexa 488
CD144+,
of C-KIT+CD45-
CD144-PE
CXCR4+,
of C-KIT+CD45-
CXCR4-BV421
SSEA3+, of
SSEA4+CD34-
SSEA3-Alexa 488
C-KIT+, of
SSEA4+CD34-
C-KIT-APC
SSEA4+CD34-,
of C-KIT+CD45-
SSEA4+CD34+,
of C-KIT+CD45-
SSEA4-PE
CD34-FITC
b
SSC
FSC
7-AAD-
7-AAD
MP
SP
Hoechst Blue
Debris (excluded
before calcuation of
percentages)
Hoechst Red
CD45-,
of SP
CD45+, of SP
CD45-PE or
CD45-FITC
C-KIT+,
of SP, CD45-
C-KIT-APC
C-KIT+,
of SP, CD45+
C-KIT-APC
SSEA-4+,
of SP, CD45-
SSEA-4-PE
SSEA-4+,
of SP, CD45+
SSEA-4-PE

S2 Fig. Gating strategy for all cell populations

Illustration of gating strategy by combination of plots from several experiments and multiple cell tubes. Please note that for some experiments, other flourochrome combinations were used in order to enable co-staining with other antibodies. a) Gating strategy for cell surface markers. DAPI could only be included for fixed, semi-permeable, cells. For non-fixated cells, debris was excluded by FSC vs SSC. b) Gating strategy for SP analysis. Debris was always excluded based on Hoechst staining (gate in lower-left corner, third plot from the left) before calculating the SP percentage of all cells.
