# Supplementary material for: Human intracardiac SSEA4+CD34- cells show features of cycling, immature cardiomyocytes and are distinct from Side Population and C-kit+CD45- cells
Source: PLoS One. 2022 Jun 16;17(6):e0269985. doi: 10.1371/journal.pone.0269985 (PMC9202910; doi:10.1371/journal.pone.0269985)
Supplement: S3 Fig — Complete representative set of plots of SSEA4 vs CD34 stainings and corresponding isotypic controls for one failing (a) and one donor heart (b), respectively. Percentages of SSEA4+CD34- cells are noted, without subtraction of isotypic controls. For isotypic controls, percentages are shown for the quadrant corresponding to the SSEA4+CD34- population. Panels to the left constitute isotypic controls. Panels to the right constitute stainings of SSEA4 vs CD34. (PDF) [file pone.0269985.s003.pdf]

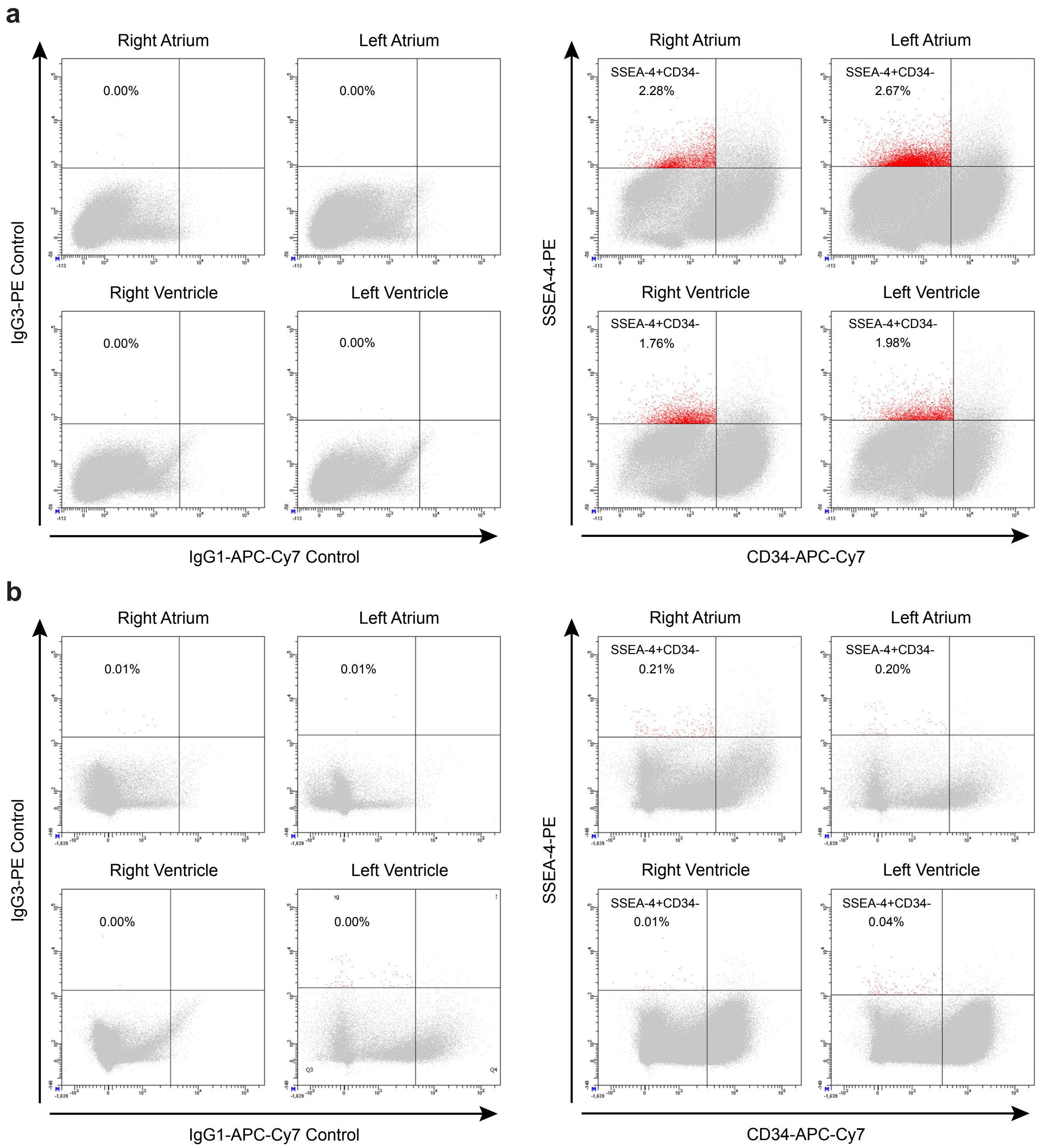


**S3 Fig. SSEA4 and CD34 expression in failing and non-failing hearts**

Complete representative set of plots of SSEA4 vs CD34 stainings and corresponding isotypic controls for one failing (a) and one donor heart (b), respectively. Percentages of SSEA4+CD34- cells are noted, without subtraction of isotypic controls. For isotypic controls, percentages are shown for the quadrant corresponding to the SSEA4+CD34- population. Panels to the left constitute isotypic controls. Panels to the right constitute stainings of SSEA4 vs CD34.
