# Supplementary material for: Human intracardiac SSEA4+CD34- cells show features of cycling, immature cardiomyocytes and are distinct from Side Population and C-kit+CD45- cells
Source: PLoS One. 2022 Jun 16;17(6):e0269985. doi: 10.1371/journal.pone.0269985 (PMC9202910; doi:10.1371/journal.pone.0269985)
Supplement: S4 Fig — Complete representative set of plots of C-kit vs CD45 stainings and corresponding isotypic controls for one failing (a) and one donor heart (b), respectively. Percentages of C-kit+CD45- cells are noted, without subtraction of isotypic controls. For isotypic controls, percentages are shown for the quadrant corresponding to the C-kit+CD45- population. Panels to the left constitute isotypic controls. Panels to the right constitute stainings of C-kit vs CD45. (PDF) [file pone.0269985.s004.pdf]

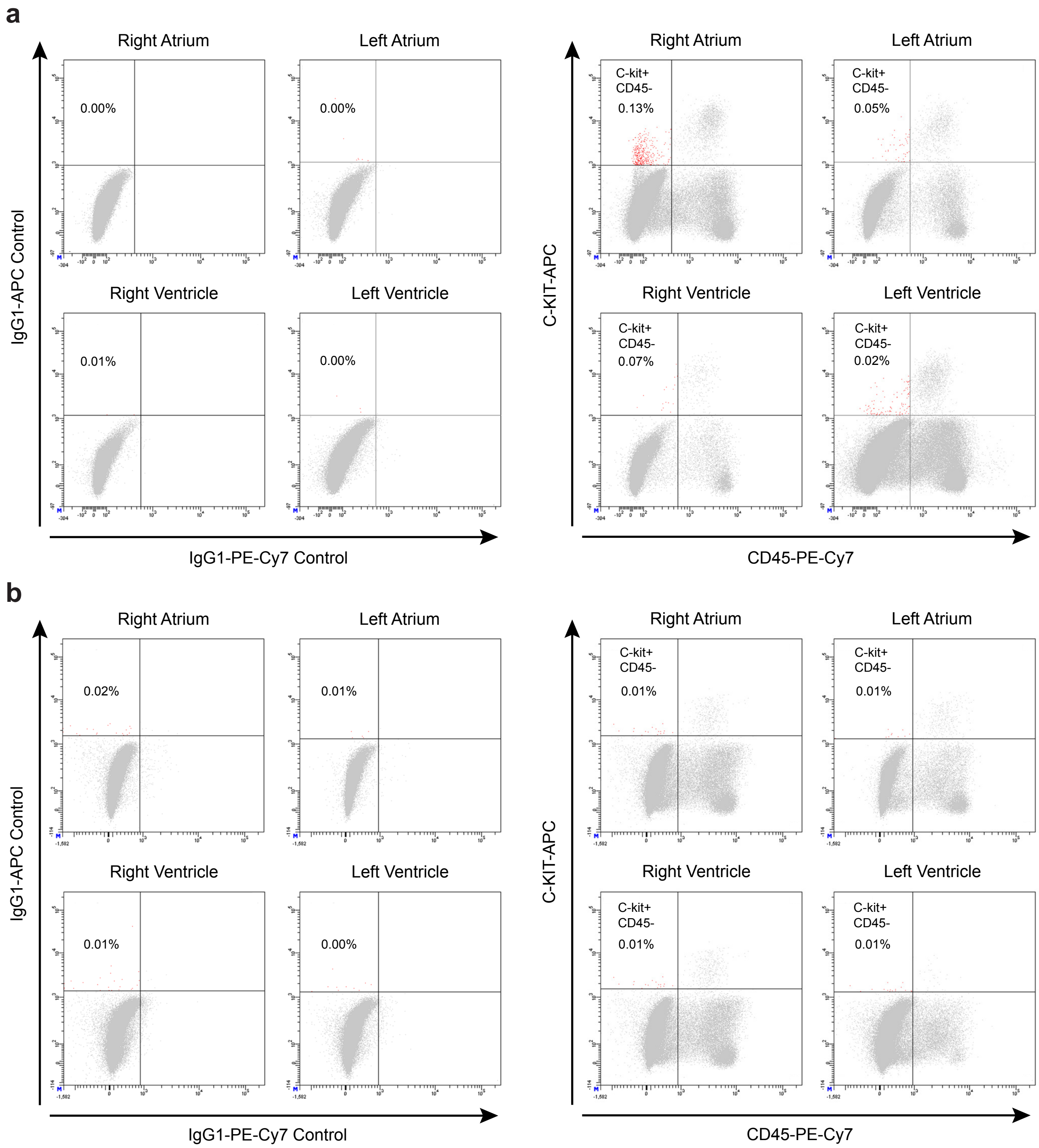

S4 Fig. C-kit and CD45 expression in failing and non-failing hearts

Complete representative set of plots of C-kit vs CD45 stainings and corresponding isotypic controls for one failing (a) and one donor heart (b), respectively. Percentages of C-kit+CD45- cells are noted, without subtraction of isotypic controls. For isotypic controls, percentages are shown for the quadrant corresponding to the C-kit+CD45- population. Panels to the left constitute isotypic controls. Panels to the right constitute stainings of C-kit vs CD45.
