# Supplementary material for: Human intracardiac SSEA4+CD34- cells show features of cycling, immature cardiomyocytes and are distinct from Side Population and C-kit+CD45- cells
Source: PLoS One. 2022 Jun 16;17(6):e0269985. doi: 10.1371/journal.pone.0269985 (PMC9202910; doi:10.1371/journal.pone.0269985)
Supplement: S5 Fig — Complete representative set of plots of SP stainings including all inhibitors, for one failing heart. Percentage of SP cells (without correction for the residual positive cells in the verapamil treated control sample) is noted for each plot. (PDF) [file pone.0269985.s005.pdf]

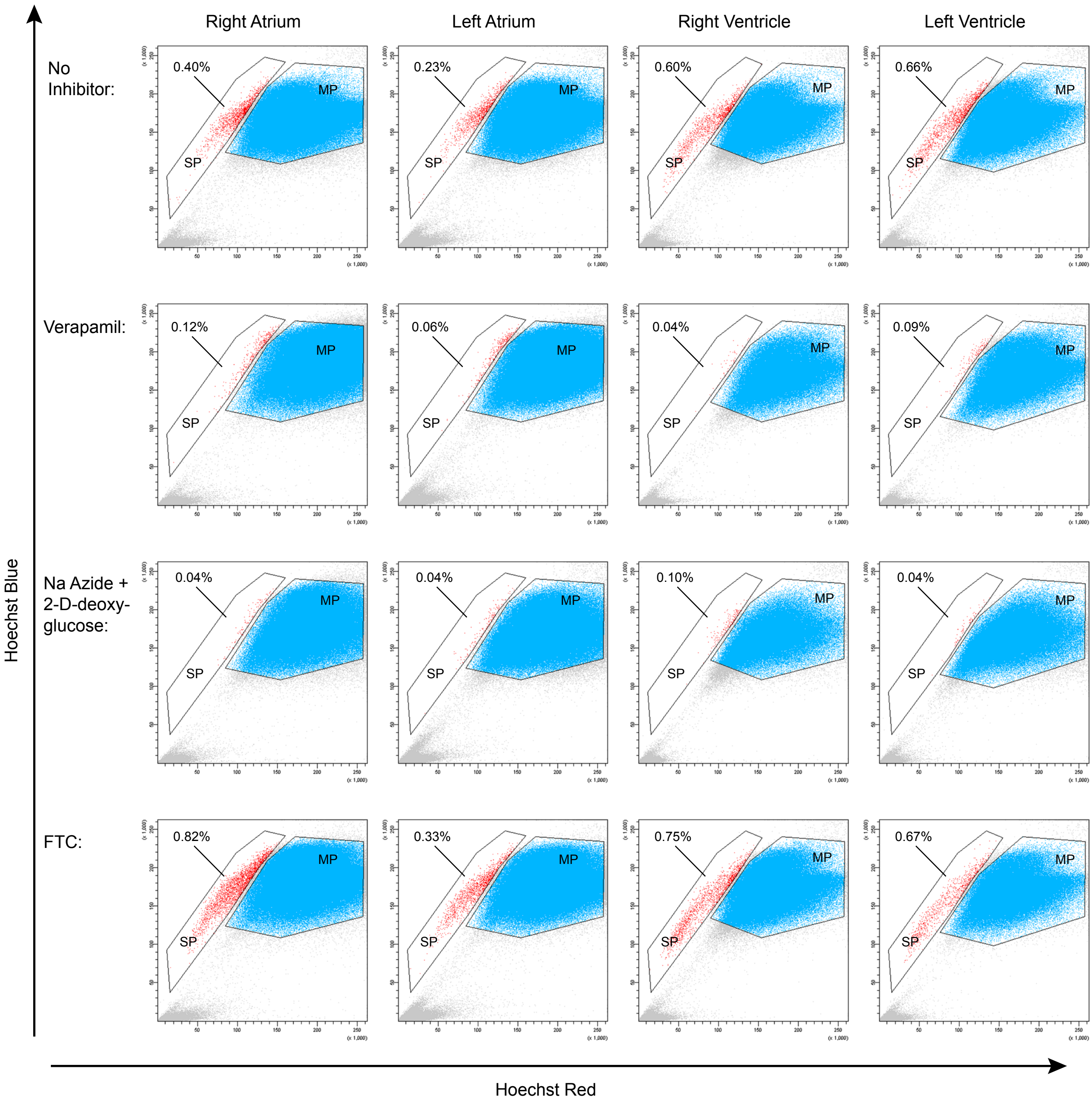

S5 Fig. Identification of Side Population cells in the failing heart

Complete representative set of plots of SP stainings including all inhibitors, for one failing heart. Percentage of SP cells (without correction for the residual positive cells in the verapamil treated control sample) is noted for each plot.
