# Supplementary material for: Human intracardiac SSEA4+CD34- cells show features of cycling, immature cardiomyocytes and are distinct from Side Population and C-kit+CD45- cells
Source: PLoS One. 2022 Jun 16;17(6):e0269985. doi: 10.1371/journal.pone.0269985 (PMC9202910; doi:10.1371/journal.pone.0269985)
Supplement: S6 Fig — Complete representative set of plots of SP stainings including all inhibitors, for one non-failing donor heart. Percentage of SP cells (without correction for the residual positive cells in the verapamil treated control sample) is noted for each plot. Please note that for this experiment, due to high percentage of debris (incompletely lysed erythrocytes), a live gate was used when collecting data for some of the samples. For these samples, debris in the lower left corner of the plots was excluded. This did not impact sorting or calculation of percentages, as debris in the lower left corner of the SP plots always was excluded by gating strategy (see also S1 Fig) before calculation of SP percentages. (PDF) [file pone.0269985.s006.pdf]

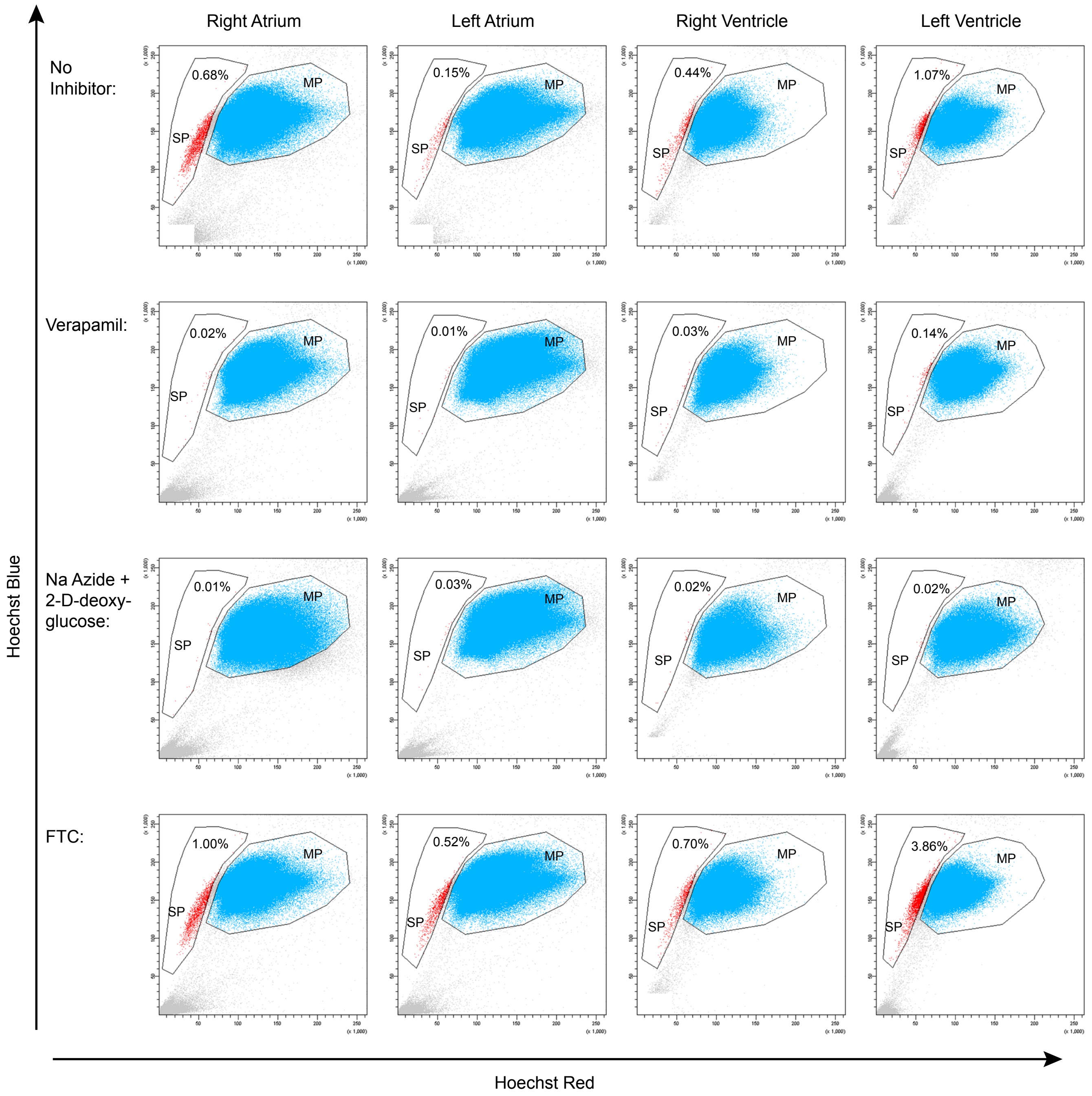

S6 Fig. Identification of Side Population cells in the non-failing heart

Complete representative set of plots of SP stainings including all inhibitors, for one non-failing donor heart. Percentage of SP cells (without correction for the residual positive cells in the verapamil treated control sample) is noted for each plot. Please note that for this experiment, due to high percentage of debris (incompletely lysed erythrocytes), a live gate was used when collecting data for some of the samples. For these samples, debris in the lower left corner of the plots was excluded. This did not impact sorting or calculation of percentages, as debris in the lower left corner of the SP plots always was excluded by gating strategy (see also S1 Fig) before calculation of SP percentages.
