# Supplementary material for: Human intracardiac SSEA4+CD34- cells show features of cycling, immature cardiomyocytes and are distinct from Side Population and C-kit+CD45- cells
Source: PLoS One. 2022 Jun 16;17(6):e0269985. doi: 10.1371/journal.pone.0269985 (PMC9202910; doi:10.1371/journal.pone.0269985)
Supplement: S7 Fig — All four cell populations were included in an unsupervised PCA model as well as an OPLS-DA model to predict population identity based on gene expression patterns. The PCA model included five significant principal components (a), and the OPLS-DA model included three significant predictive components (b). Cumulative R2X and R2Y were calculated to measure the explained variation of gene expression and population identity, respectively. Cumulative Q2 was calculated to measure the robustness of the models, using cross-validation. (PDF) [file pone.0269985.s007.pdf]

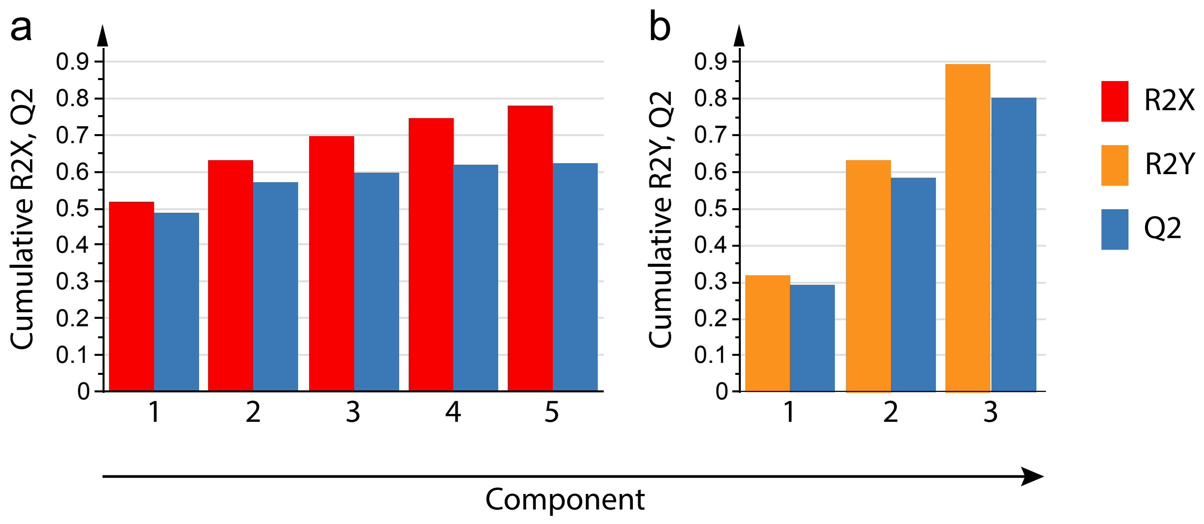

S7 Fig. PCA and OPLS-DA model characteristics

All four cell populations were included in an unsupervised PCA model as well as an OPLS-DA model to predict population identity based on gene expression patterns. The PCA model included five significant principal components (a), and the OPLS-DA model included three significant predictive components (b). Cumulative R2X and R2Y were calculated to measure the explained variation of gene expression and population identity, respectively. Cumulative Q2 was calculated to measure the robustness of the models, using cross-validation.
