# Supplementary material for: Human intracardiac SSEA4+CD34- cells show features of cycling, immature cardiomyocytes and are distinct from Side Population and C-kit+CD45- cells
Source: PLoS One. 2022 Jun 16;17(6):e0269985. doi: 10.1371/journal.pone.0269985 (PMC9202910; doi:10.1371/journal.pone.0269985)
Supplement: S8 Fig — All four cell populations were included in the unsupervised PCA model (n = 44). a) Cell populations visualized in a score plot of the third and fourth principal components (PC). While there was no distinct clustering of the respective populations, a subset of SP CD45- and SP CD45+ samples tended to cluster together based on the third PC (dashed circle). b-h) Gene expression patterns, demonstrated by loading plots. The cluster of SP CD45- and SP CD45+ samples was associated with high expression of VEGF, BMP and TGF-β markers (dashed circles). Genes have been color-and symbol-coded based on the corresponding gene annotation, as noted to the right of each figure. To improve visualization, some genes are included in more than one panel due to multiple annotations. (PDF) [file pone.0269985.s008.pdf]

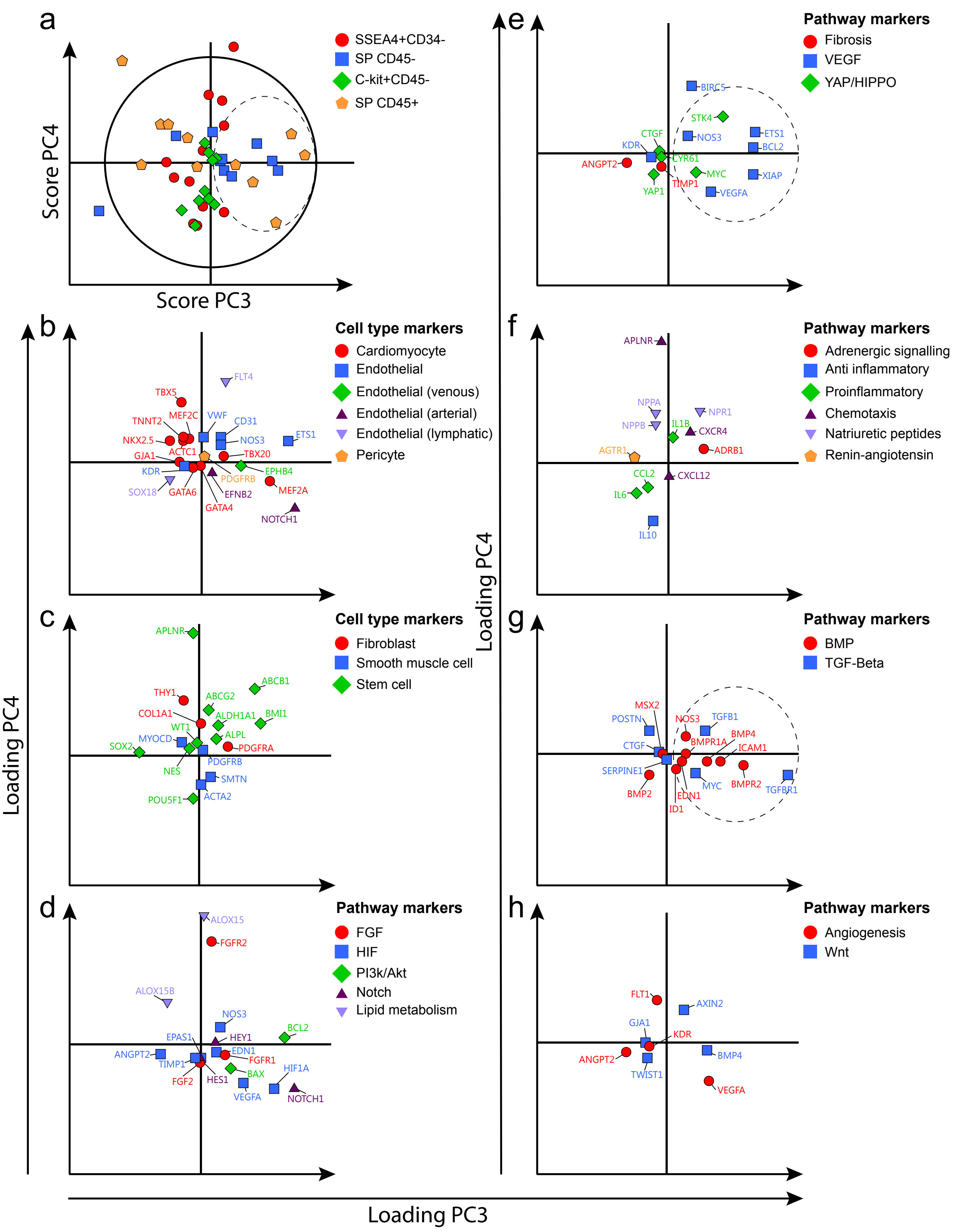
a
SSEA4+CD34-
SP CD45-
C-kit+CD45-
SP CD45+
Score PC4
Score PC3
b
Cell type markers
Cardiomyocyte
Endothelial
Endothelial (venous)
Endothelial (arterial)
Endothelial (lymphatic)
Pericyte
c
Cell type markers
Fibroblast
Smooth muscle cell
Stem cell
d
Pathway markers
FGF
HIF
PI3k/Akt
Notch
Lipid metabolism
e
Pathway markers
Fibrosis
VEGF
YAP/HIPPO
f
Pathway markers
Adrenergic signalling
Anti inflammatory
Proinflammatory
Chemotaxis
Natriuretic peptides
Renin-angiotensin
g
Pathway markers
BMP
TGF-Beta
h
Pathway markers
Angiogenesis
Wnt
Loading PC4
Loading PC3

S8 Fig. Gene expression patterns of intracardiac SSEA4+CD34-, SP CD45-, SP CD45+ and C-kit+CD45- cells, as visualized by the third and fourth principal components

All four cell populations were included in the unsupervised PCA model (n=44). a) Cell populations visualized in a score plot of the third and fourth principal components (PC). While there was no distinct clustering of the respective populations, a subset of SP CD45- and SP CD45+ samples tended to cluster together based on the third PC (dashed circle). b-h) Gene expression patterns, demonstrated by loading plots. The cluster of SP CD45- and SP CD45+ samples was associated with high expression of VEGF, BMP and TGFβ markers (dashed circles). Genes have been color- and symbol-coded based on the corresponding gene annotation, as noted to the right of each figure. To improve visualization, some genes are included in more than one panel due to multiple annotations.
