# Supplementary material for: Human intracardiac SSEA4+CD34- cells show features of cycling, immature cardiomyocytes and are distinct from Side Population and C-kit+CD45- cells
Source: PLoS One. 2022 Jun 16;17(6):e0269985. doi: 10.1371/journal.pone.0269985 (PMC9202910; doi:10.1371/journal.pone.0269985)
Supplement: S9 Fig — To determine differentially expressed genes by SSEA4+CD34- cells, MP was used as reference population (n = 12). Significantly differentially expressed genes at an FDR of < 5% are included in the heatmap. Several pathway markers, including BCL2, BAX, YAP1, NPPA, NPPB, IL6, IL10, CXCR4, ADRB1 were expressed at higher levels in SSEA4+CD34- cells (a-c). The majority of BMP markers were however expressed at lower levels (d). The heat color scale has been centered with a mean of 0 and a standard deviation of 1, for each gene. Hierarchical clustering resulted in separation between SSEA4+CD34- and MP cells. Genes and populations have been color-coded based on the corresponding annotations, as noted to the right of each figure. To improve visualization, some genes are included in more than one panel due to multiple annotations. HF = Heart failure patient, Don = Donor. (PDF) [file pone.0269985.s009.pdf]

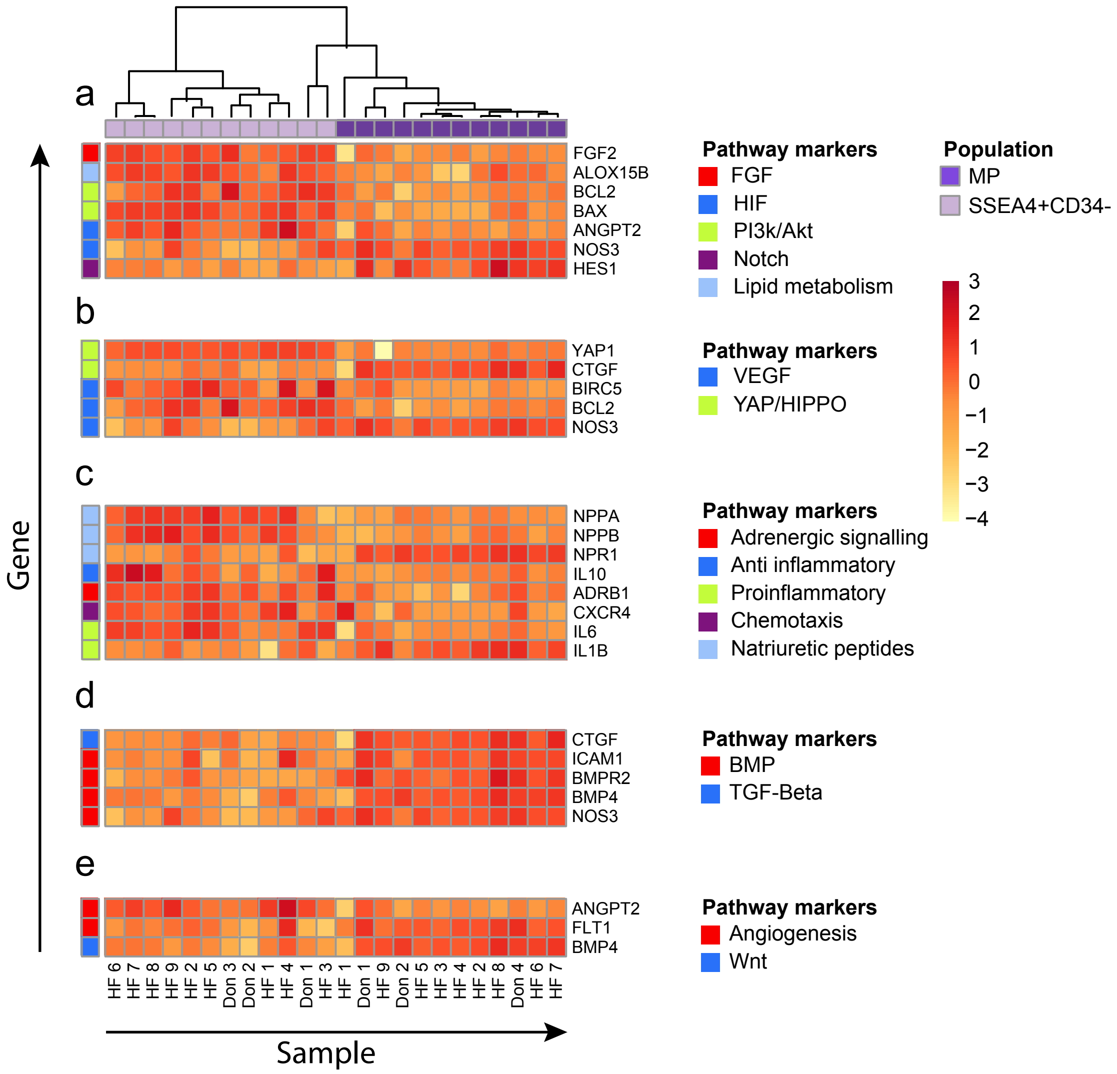

S9 Fig. Differentially expressed pathway marker genes by SSEA4+CD34- cells

To determine differentially expressed genes by SSEA4+CD34- cells, MP was used as reference population (n=12). Significantly differentially expressed genes at an FDR of < 5% are included in the heatmap. Several pathway markers, including *BCL2*, *BAX*, *YAP1*, *NPPA*, *NPPB*, *IL6*, *IL10*, *CXCR4*, *ADRB1* were expressed at higher levels in SSEA4+CD34- cells (a-c). The majority of BMP markers were however expressed at lower levels (d). The heat color scale has been centered with a mean of 0 and a standard deviation of 1, for each gene. Hierarchical clustering resulted in separation between SSEA4+CD34- and MP cells. Genes and populations have been color-coded based on the corresponding annotations, as noted to the right of each figure. To improve visualization, some genes are included in more than one panel due to multiple annotations. HF = Heart failure patient, Don = Donor.
