# Supplementary material for: Human intracardiac SSEA4+CD34- cells show features of cycling, immature cardiomyocytes and are distinct from Side Population and C-kit+CD45- cells
Source: PLoS One. 2022 Jun 16;17(6):e0269985. doi: 10.1371/journal.pone.0269985 (PMC9202910; doi:10.1371/journal.pone.0269985)
Supplement: S10 Fig — To determine differentially expressed genes by SP CD45- cells, MP was used as reference population (n = 11). Significantly differentially expressed genes at an FDR of < 5% are included in the heatmap. Several pathway markers, including NPPA, AGTR1 and IL1B were expressed at lower levels in SP CD45- cells (c). YAP1 on the other hand was expressed at higher levels in SP CD45- cells (b). The heat color scale has been centered with a mean of 0 and a standard deviation of 1, for each gene. Hierarchical clustering resulted in separation between SP CD45- and MP cells in heatmaps for which the number of differentially expressed genes was sufficient for hierarchical clustering (approximately 4–5 genes, a-b). Genes and populations have been color-coded based on the corresponding annotations, as noted to the right of each figure. To improve visualization, some genes are included in more than one panel due to multiple annotations. HF = Heart failure patient, Don = Donor. (PDF) [file pone.0269985.s010.pdf]

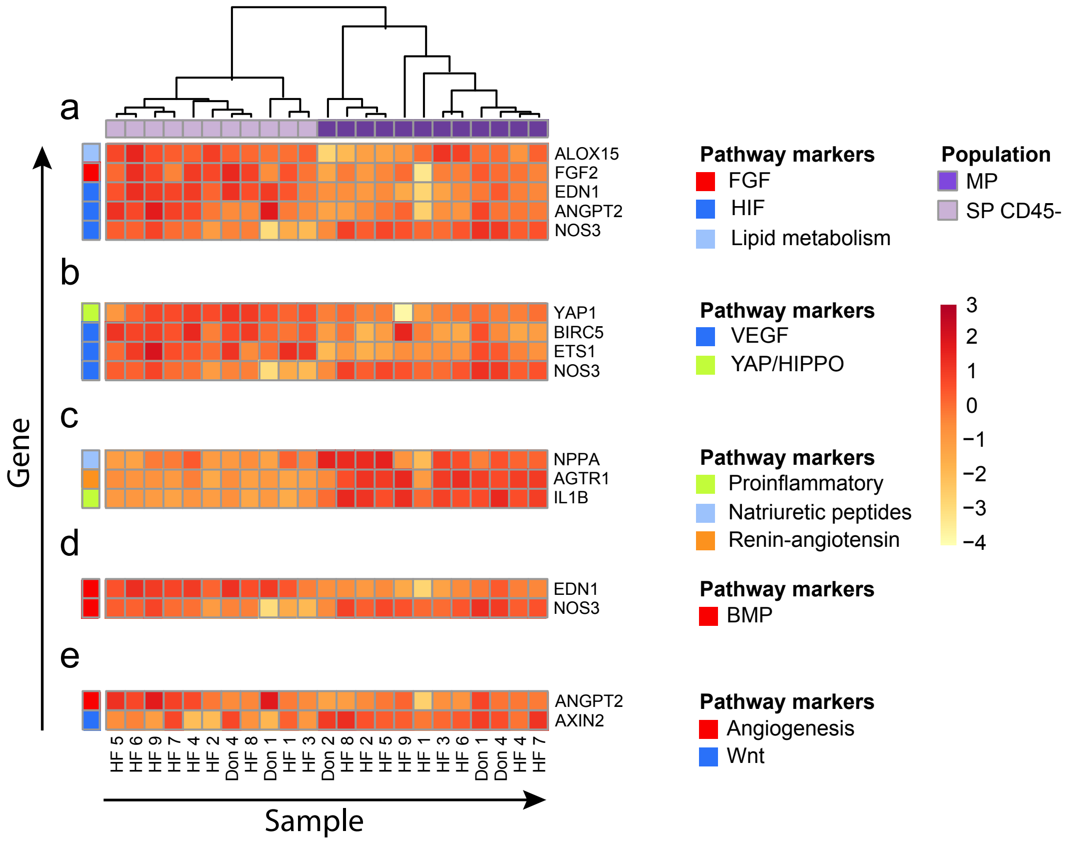

S10 Fig. Differentially expressed pathway marker genes by SP CD45- cells

To determine differentially expressed genes by SP CD45- cells, MP was used as reference population (n=11). Significantly differentially expressed genes at an FDR of < 5% are included in the heatmap. Several pathway markers, including *NPPA*, *AGTR1* and *IL1B* were expressed at lower levels in SP CD45- cells (c). *YAP1* on the other hand was expressed at higher levels in SP CD45- cells (b). The heat color scale has been centered with a mean of 0 and a standard deviation of 1, for each gene. Hierarchical clustering resulted in separation between SP CD45- and MP cells in heatmaps for which the number of differentially expressed genes was sufficient for hierarchical clustering (approximately 4-5 genes, a-b). Genes and populations have been color-coded based on the corresponding annotations, as noted to the right of each figure. To improve visualization, some genes are included in more than one panel due to multiple annotations. HF = Heart failure patient, Don = Donor.
