# Supplementary material for: Human intracardiac SSEA4+CD34- cells show features of cycling, immature cardiomyocytes and are distinct from Side Population and C-kit+CD45- cells
Source: PLoS One. 2022 Jun 16;17(6):e0269985. doi: 10.1371/journal.pone.0269985 (PMC9202910; doi:10.1371/journal.pone.0269985)
Supplement: S11 Fig — To determine differentially expressed genes by SP CD45+ cells, MP was used as reference population (n = 11). Significantly differentially expressed genes at an FDR of < 5% are included in the heatmap. Most differentially expressed markers were expressed at lower levels in SP CD45+ cells, with a few exceptions–such as ADRB1 and CXCR4 (c). The heat color scale has been centered with a mean of 0 and a standard deviation of 1, for each gene. Hierarchical clustering resulted in separation between SP CD45+ and MP cells. Genes and populations have been color-coded based on the corresponding annotations, as noted to the right of each figure. To improve visualization, some genes are included in more than one panel due to multiple annotations. HF = Heart failure patient, Don = Donor. (PDF) [file pone.0269985.s011.pdf]

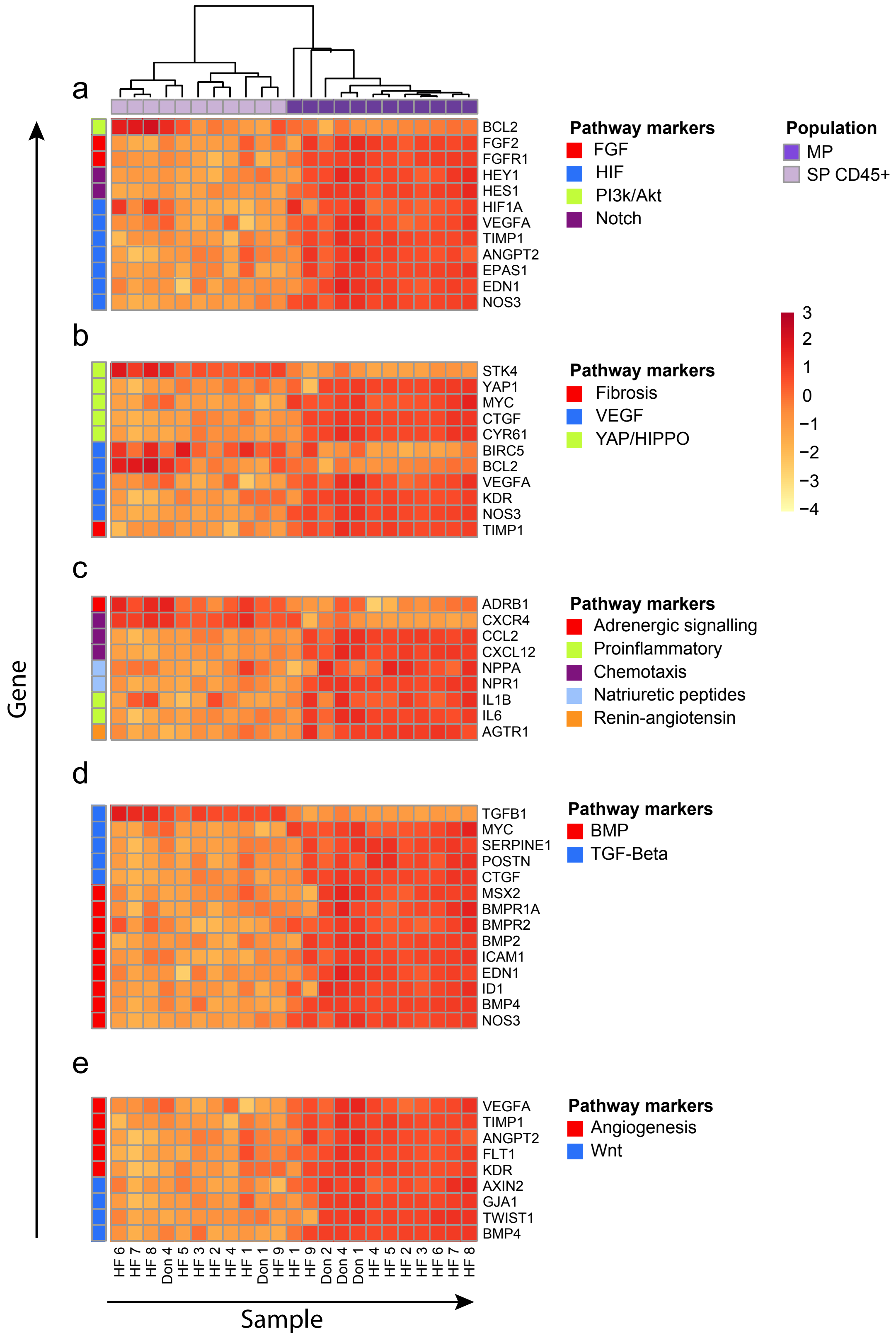

a
b
c
d
e
Pathway markers
FGF
HIF
PI3k/Akt
Notch
Population
MP
SP CD45+
BCL2
FGF2
FGFR1
HEY1
HES1
HIF1A
VEGFA
TIMP1
ANGPT2
EPAS1
EDN1
NOS3
Pathway markers
Fibrosis
VEGF
YAP/HIPPO
STK4
YAP1
MYC
CTGF
CYR61
BIRC5
BCL2
VEGFA
KDR
NOS3
TIMP1
3
2
1
0
−1
−2
−3
−4
Pathway markers
Adrenergic signalling
Proinflammatory
Chemotaxis
Natriuretic peptides
Renin-angiotensin
ADRB1
CXCR4
CCL2
CXCL12
NPPA
NPR1
IL1B
IL6
AGTR1
Pathway markers
BMP
TGF-Beta
TGFB1
MYC
SERPINE1
POSTN
CTGF
MSX2
BMPR1A
BMPR2
BMP2
ICAM1
EDN1
ID1
BMP4
NOS3
Pathway markers
Angiogenesis
Wnt
VEGFA
TIMP1
ANGPT2
FLT1
KDR
AXIN2
GJA1
TWIST1
BMP4
HF 6
HF 7
HF 8
Don 4
HF 5
HF 3
HF 2
HF 4
HF 1
Don 1
HF 9
HF 1
HF 9
Don 2
Don 4
Don 1
HF 4
HF 5
HF 2
HF 3
HF 6
HF 7
HF 8
Gene
Sample

S11 Fig. Differentially expressed pathway marker genes by SP CD45+ cells

To determine differentially expressed genes by SP CD45+ cells, MP was used as reference population (n=11). Significantly differentially expressed genes at an FDR of < 5% are included in the heatmap. Most differentially expressed markers were expressed at lower levels in SP CD45+ cells, with a few exceptions – such as *ADRB1* and *CXCR4* (c). The heat color scale has been centered with a mean of 0 and a standard deviation of 1, for each gene. Hierarchical clustering resulted in separation between SP CD45+ and MP cells. Genes and populations have been color-coded based on the corresponding annotations, as noted to the right of each figure. To improve visualization, some genes are included in more than one panel due to multiple annotations. HF = Heart failure patient, Don = Donor.
