# Supplementary material for: Human intracardiac SSEA4+CD34- cells show features of cycling, immature cardiomyocytes and are distinct from Side Population and C-kit+CD45- cells
Source: PLoS One. 2022 Jun 16;17(6):e0269985. doi: 10.1371/journal.pone.0269985 (PMC9202910; doi:10.1371/journal.pone.0269985)
Supplement: S13 Fig — SSEA4+CD34- cells isolated from failing hearts as well as donor hearts were included in an OPLS-DA model to predict presence of heart failure based on gene expression patterns. The model included one significant predictive component and one orthogonal component. Cumulative R2Y was calculated to measure the explained variation of heart failure identity. Cumulative Q2 was calculated to measure the robustness of the model, using cross-validation. (PDF) [file pone.0269985.s013.pdf]

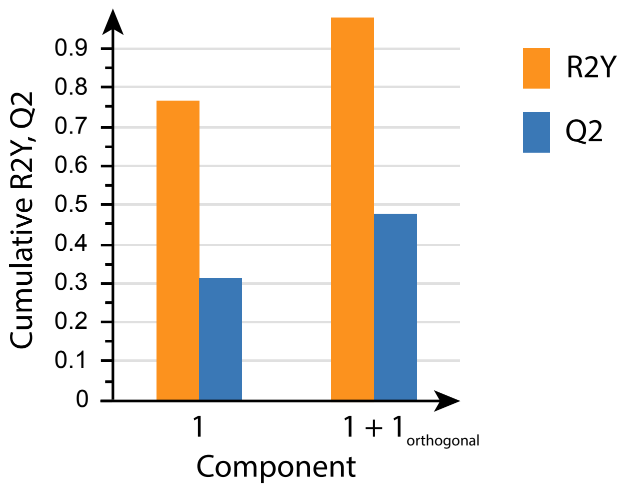

S13 Fig. OPLS-DA model characteristics

SSEA4+CD34- cells isolated from failing hearts as well as donor hearts were included in an OPLS-DA model to predict presence of heart failure based on gene expression patterns. The model included one significant predictive component and one orthogonal component. Cumulative R2Y was calculated to measure the explained variation of heart failure identity. Cumulative Q2 was calculated to measure the robustness of the model, using cross-validation.
