# Supplementary material for: Human intracardiac SSEA4+CD34- cells show features of cycling, immature cardiomyocytes and are distinct from Side Population and C-kit+CD45- cells
Source: PLoS One. 2022 Jun 16;17(6):e0269985. doi: 10.1371/journal.pone.0269985 (PMC9202910; doi:10.1371/journal.pone.0269985)
Supplement: S14 Fig — a) All four cell populations of interest were included in the supervised OPLS-DA model (included independent cell population datasets = 35). Only samples isolated from failing hearts were included. Clustering of SSEA4+CD34- cells, as demonstrated by the two first OPLS-DA predictive components (PCs) in a score plot. b) Summary of fit, as demonstrated by cumulative R2Y and Q2 for the three included predictive components. c-d) Scaled and centered OPLS-DA regression coefficients with 95% confidence intervals are shown. While pathway markers were included in the OPLS-DA model, only regression coefficients corresponding to cell type markers have been included in the figure. Significant predicting variables have been marked with "*". PC = Predictive Component. (PDF) [file pone.0269985.s014.pdf]

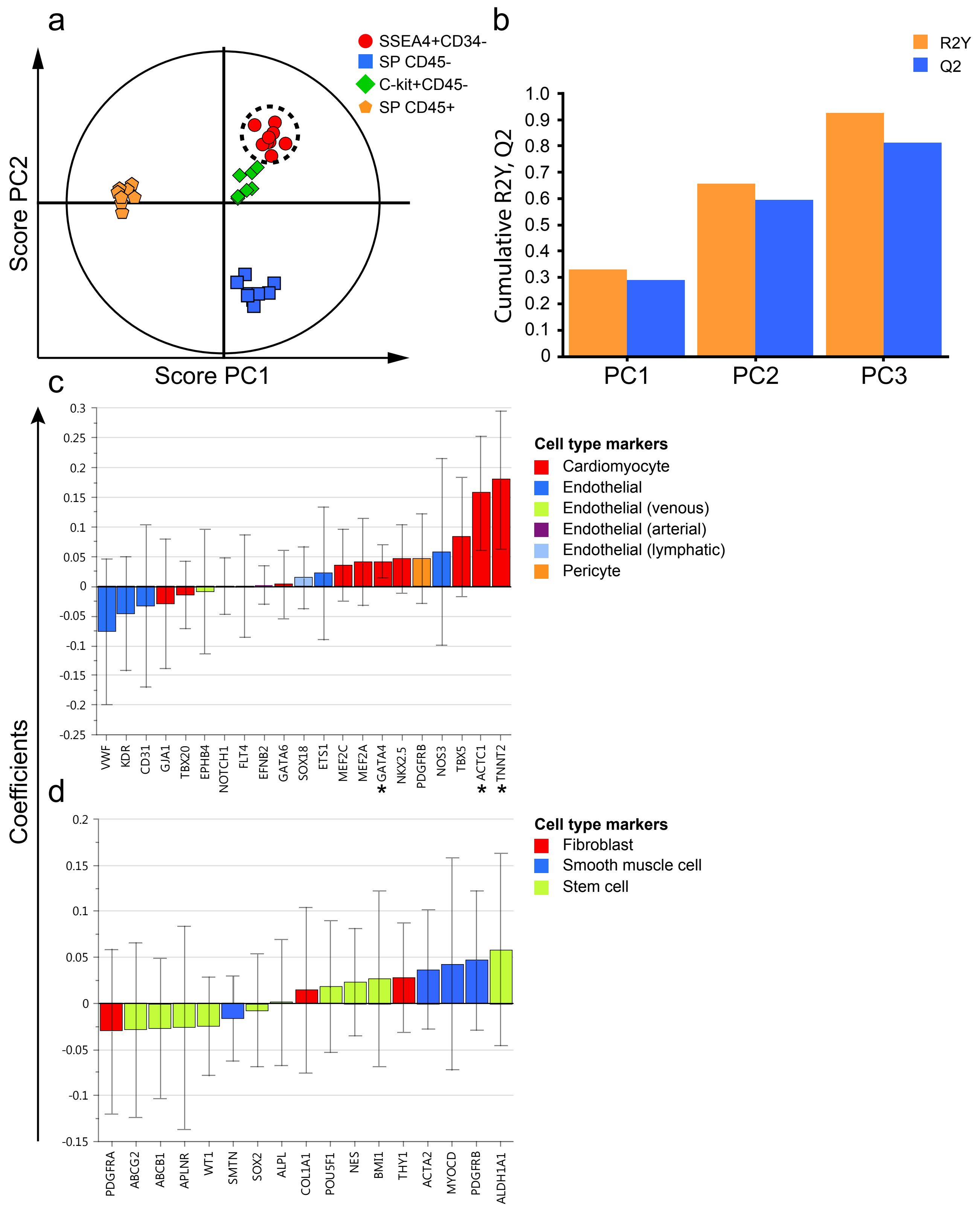
a
SSEA4+CD34-
SP CD45-
C-kit+CD45-
SP CD45+
Score PC2
Score PC1
b
R2Y
Q2
Cumulative R2Y, Q2
1.0
0.9
0.8
0.7
0.6
0.5
0.4
0.3
0.2
0.1
0
PC1
PC2
PC3
c
Coefficients
Cell type markers
Cardiomyocyte
Endothelial
Endothelial (venous)
Endothelial (arterial)
Endothelial (lymphatic)
Pericyte
VWF
KDR
CD31
GJA1
TBX20
EPHB4
NOTCH1
FLT4
EFNB2
GATA6
SOX18
ETS1
MEF2C
MEF2A
* GATA4
NKX2.5
PDGFRB
NOS3
TBX5
* ACTC1
* TNNT2
d
Cell type markers
Fibroblast
Smooth muscle cell
Stem cell
PDGFRA
ABCG2
ABCB1
APLNR
WT1
SMTN
SOX2
ALPL
COL1A1
POU5F1
NES
BMI1
THY1
ACTA2
MYOCD
PDGFRB
ALDH1A1

S14 Fig. Differentially expressed genes by SSEA4+CD34- cells in failing hearts.

a) All four cell populations of interest were included in the supervised OPLS-DA model (included independent cell population datasets = 35). Only samples isolated from failing hearts were included. Clustering of SSEA4+CD34- cells, as demonstrated by the two first OPLS-DA predictive components (PCs) in a score plot. b) Summary of fit, as demonstrated by cumulative R2Y and Q2 for the three included predictive components. c-d) Scaled and centered OPLS-DA regression coefficients with 95% confidence intervals are shown. While pathway markers were included in the OPLS-DA model, only regression coefficients corresponding to cell type markers have been included in the figure. Significant predicting variables have been marked with ”*”. PC = Predictive Component.
