# Supplementary material for: Human intracardiac SSEA4+CD34- cells show features of cycling, immature cardiomyocytes and are distinct from Side Population and C-kit+CD45- cells
Source: PLoS One. 2022 Jun 16;17(6):e0269985. doi: 10.1371/journal.pone.0269985 (PMC9202910; doi:10.1371/journal.pone.0269985)
Supplement: S15 Fig — The expression of 7 positive cell cycle regulators and 2 cell cycle inhibitors were analyzed through qPCR. SSEA4+CD34-, CD45+ and CD45- SP, C-kit+CD45- and MP cell samples from the left atrium and left ventricle of both failing and non-failing hearts were included. A mean delta CT value for each heart was calculated after correction for fixation and presence/absence of heart failure. Prior to PCA model fitting, delta CT values were transformed by adding 1, followed by log transformation, to accommodate transformation of 0 values. Three outliers were excluded based on Hotellings T2 and DmodX. A two component model with low reproducibility was obtained, as demonstrated by the low cumulative Q2 values (a). SSEA4+CD34-, MP, SP CD45- and C-kit+CD45- cells clustered together within the score plot, indicating an overall similar expression pattern of cell cycle regulators (b). SP CD45+ cells tended to cluster separately from the other cell populations. There was no clear correlation between cell population clustering and expression of cell cycle inhibitors or positive cell cycle regulators, as demonstrated by the loading plot (c). PC = Principle Component. (PDF) [file pone.0269985.s015.pdf]

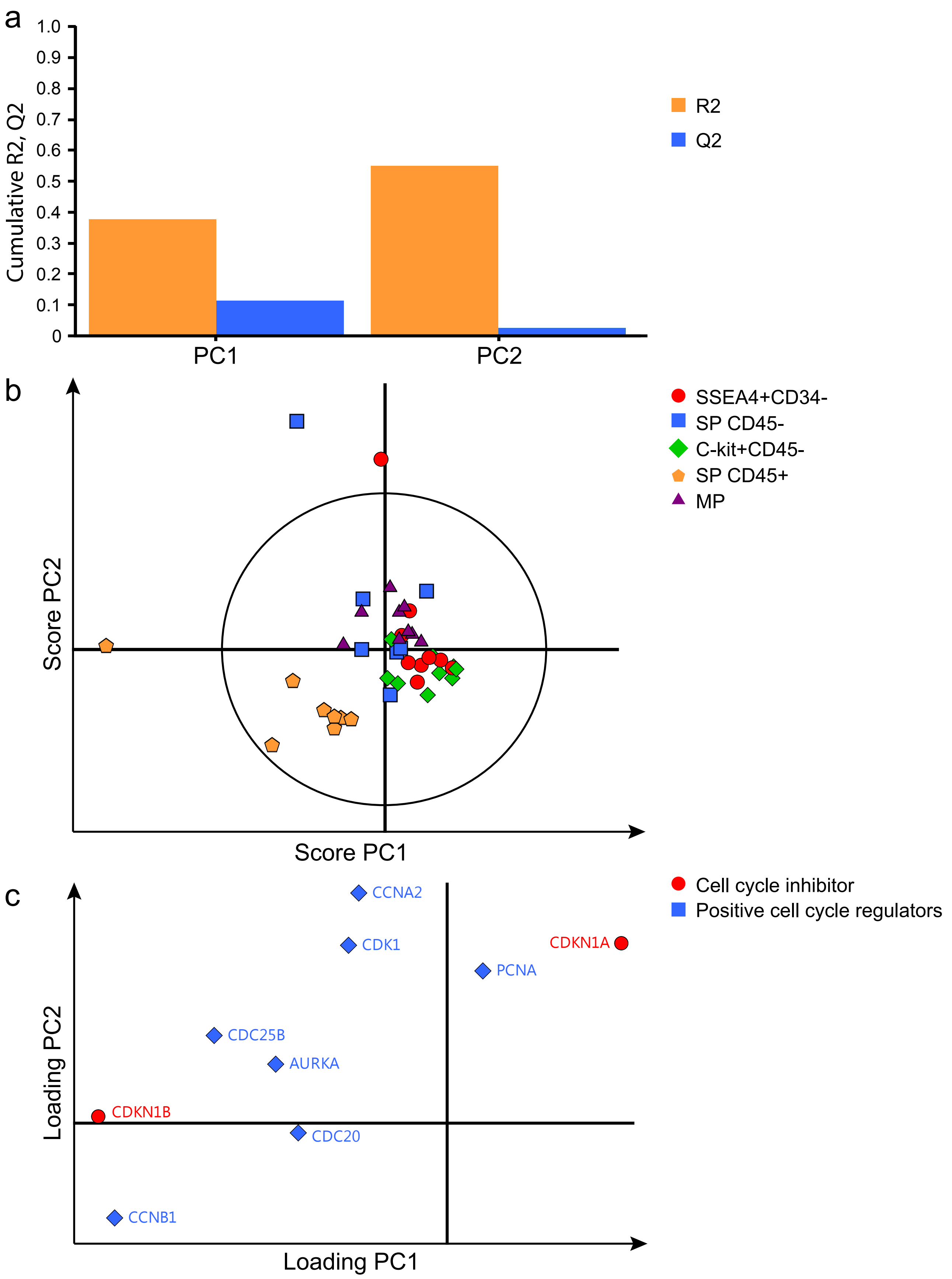
a
Cumulative R2, Q2
1.0
0.9
0.8
0.7
0.6
0.5
0.4
0.3
0.2
0.1
0
PC1
PC2
R2
Q2
b
Score PC2
Score PC1
SSEA4+CD34-
SP CD45-
C-kit+CD45-
SP CD45+
MP
c
Loading PC2
Loading PC1
CCNA2
CDK1
CDKN1A
PCNA
CDC25B
AURKA
CDKN1B
CDC20
CCNB1
Cell cycle inhibitor
Positive cell cycle regulators

S15 Fig. Expression of cell cycle regulators.

The expression of 7 positive cell cycle regulators and 2 cell cycle inhibitors were analyzed through qPCR. SSEA4+CD34 , CD45+ and CD45 SP, C kit+CD45 and MP cell samples from the left atrium and left ventricle of both failing and non-failing hearts were included. A mean delta CT value for each heart was calculated after correction for fixation and presence/absence of heart failure. Prior to PCA model fitting, delta CT values were transformed by adding 1, followed by log transformation, to accommodate transformation of 0 values. Three outliers were excluded based on Hotellings T2 and DmodX. A two component model with low reproducibility was obtained, as demonstrated by the low cumulative Q2 values (a). SSEA4+CD34 , MP, SP CD45 and C kit+CD45 cells clustered together within the score plot, indicating an overall similar expression pattern of cell cycle regulators (b). SP CD45+ cells tended to cluster separately from the other cell populations. There was no clear correlation between cell population clustering and expression of cell cycle inhibitors or positive cell cycle regulators, as demonstrated by the loading plot (c). PC = Principle Component.
