# Supplementary material for: Human intracardiac SSEA4+CD34- cells show features of cycling, immature cardiomyocytes and are distinct from Side Population and C-kit+CD45- cells
Source: PLoS One. 2022 Jun 16;17(6):e0269985. doi: 10.1371/journal.pone.0269985 (PMC9202910; doi:10.1371/journal.pone.0269985)
Supplement: S16 Fig — SP CD45- (n = 37), SP CD45+ (n = 37), C-kit+CD45- (n = 32) and MP (n = 44) cells isolated from the four different heart chambers were included in four separate OPLSDA models, predicting study participant identity. The orthogonal score plots were used to analyze the clustering of samples based on heart chamber identity. SP CD45- and SP CD45+ samples demonstrated no distinct clustering. C-kit+CD45- cells isolated from right atria tended to cluster separately. MP samples isolated from atria and ventricles clustered separately. (PDF) [file pone.0269985.s016.pdf]

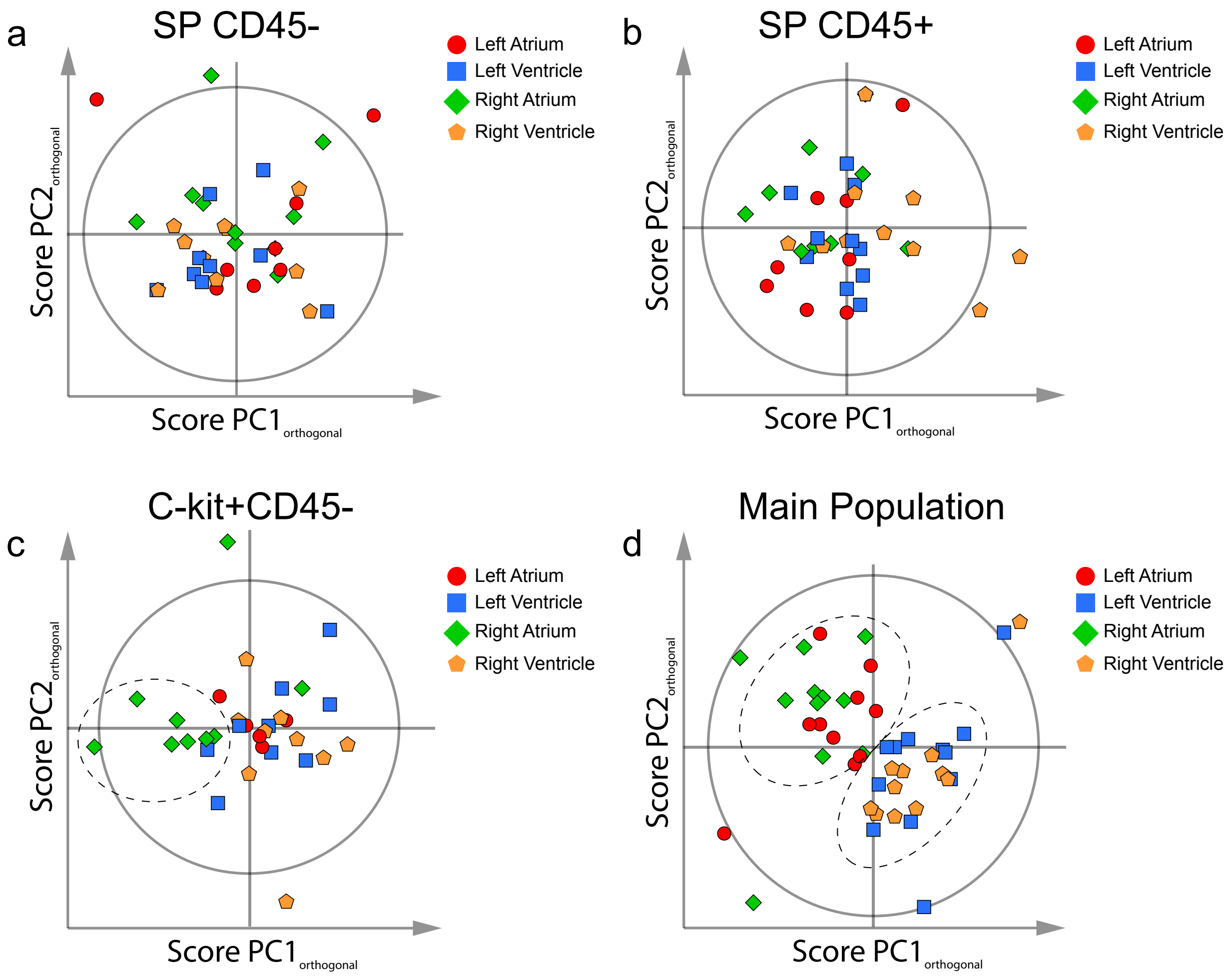

S16 Fig. Clustering of samples based on heart chamber identity

SP CD45- (n=37), SP CD45+ (n=37), C-kit+CD45- (n=32) and MP (n=44) cells isolated from the four different heart chambers were included in four separate OPLS-DA models, predicting study participant identity. The orthogonal score plots were used to analyze the clustering of samples based on heart chamber identity. SP CD45- and SP CD45+ samples demonstrated no distinct clustering. C-kit+CD45- cells isolated from right atria tended to cluster separately. MP samples isolated from atria and ventricles clustered separately.
