# Supplementary material for: Human intracardiac SSEA4+CD34- cells show features of cycling, immature cardiomyocytes and are distinct from Side Population and C-kit+CD45- cells
Source: PLoS One. 2022 Jun 16;17(6):e0269985. doi: 10.1371/journal.pone.0269985 (PMC9202910; doi:10.1371/journal.pone.0269985)
Supplement: S17 Fig — C-kit+CD45- cells isolated from the four different heart chambers were included in an OPLS-DA model (n = 32), predicting study participant identity. a) C-kit+CD45- cells isolated from right atrium tended to cluster separately as shown by the two first orthogonal principal components (PC) in a score plot. b-h) Gene expression patterns, demonstrated by orthogonal loading plots. C-kit+CD45- isolated from right atria expressed high levels of endothelial markers as well as markers involved in HIF, YAP/HIPPO, chemotactic, BMP and pro-angiogenic signaling. Genes have been color- and symbol-coded based on the corresponding gene annotation, as noted to the right of each figure. To improve visualization, some genes are included in more than one panel due to multiple annotations. (PDF) [file pone.0269985.s017.pdf]

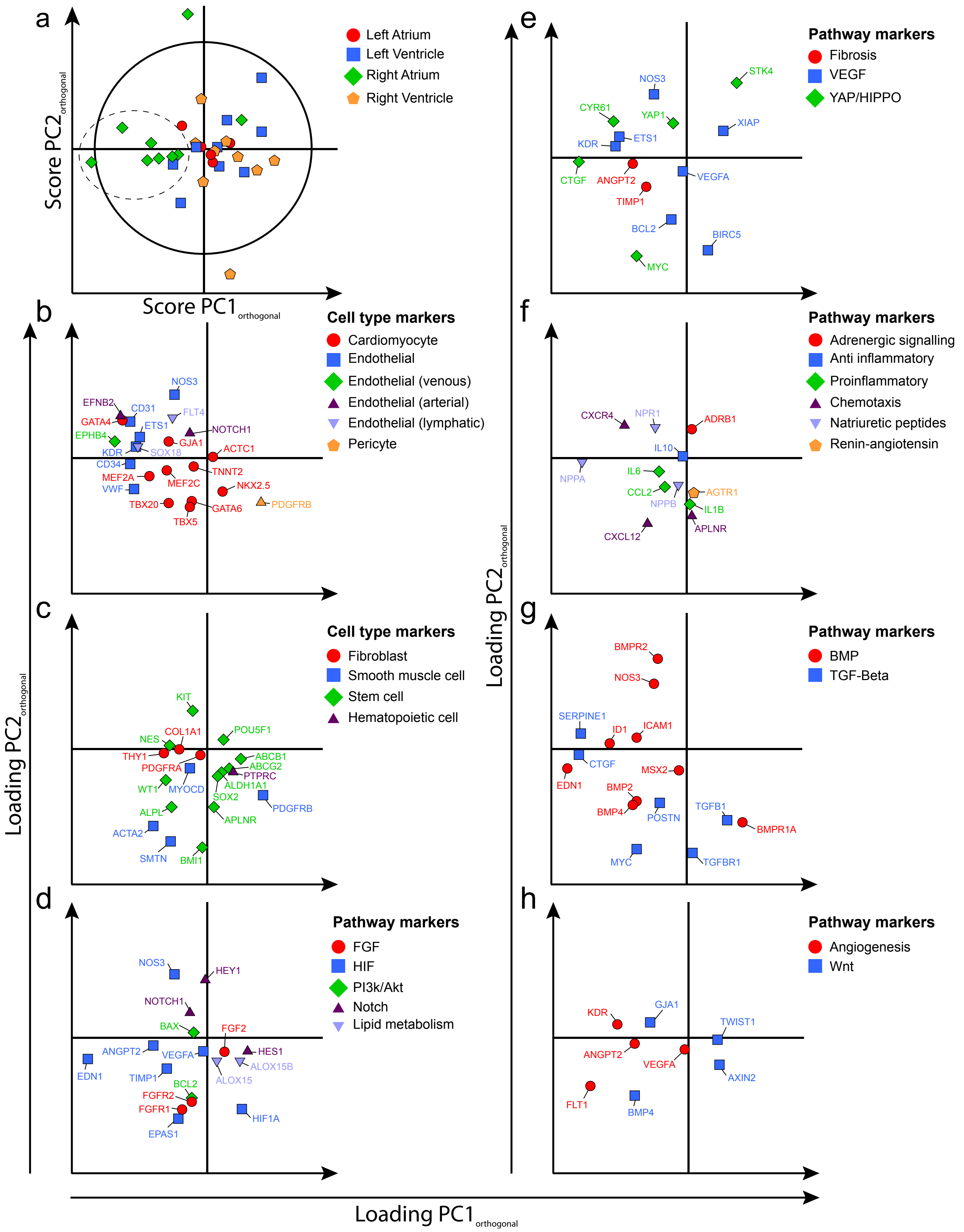

a
Left Atrium
Left Ventricle
Right Atrium
Right Ventricle
Score PC2 orthogonal
Score PC1 orthogonal
b
Cell type markers
Cardiomyocyte
Endothelial
Endothelial (venous)
Endothelial (arterial)
Endothelial (lymphatic)
Pericyte
NOS3
EFNB2
CD31
FLT4
GATA4
ETS1
NOTCH1
EPHB4
GJA1
KDR
SOX18
ACTC1
CD34
TNNT2
MEF2A
MEF2C
VWF
NKX2.5
TBX20
GATA6
PDGFRB
TBX5
c
Cell type markers
Fibroblast
Smooth muscle cell
Stem cell
Hematopoietic cell
KIT
POU5F1
COL1A1
NES
THY1
ABCB1
PDGFRA
ABCG2
PTPRC
WT1
MYOCD
ALDH1A1
SOX2
PDGFRB
ALPL
APLNR
ACTA2
SMTN
BMI1
d
Pathway markers
FGF
HIF
PI3k/Akt
Notch
Lipid metabolism
NOS3
HEY1
NOTCH1
BAX
FGF2
ANGPT2
VEGFA
HES1
EDN1
TIMP1
ALOX15B
ALOX15
BCL2
FGFR2
FGFR1
HIF1A
EPAS1
Loading PC2 orthogonal
Loading PC1 orthogonal
e
Pathway markers
Fibrosis
VEGF
YAP/HIPPO
STK4
NOS3
CYR61
YAP1
XIAP
KDR
ETS1
CTGF
ANGPT2
VEGFA
TIMP1
BCL2
BIRC5
MYC
f
Pathway markers
Adrenergic signalling
Anti inflammatory
Proinflammatory
Chemotaxis
Natriuretic peptides
Renin-angiotensin
CXCR4
NPR1
ADRB1
IL10
NPPA
IL6
CCL2
AGTR1
NPPB
IL1B
APLNR
CXCL12
g
Pathway markers
BMP
TGF-Beta
BMPR2
NOS3
SERPINE1
ICAM1
ID1
CTGF
MSX2
EDN1
BMP2
TGFB1
BMP4
POSTN
BMPR1A
MYC
TGFBR1
h
Pathway markers
Angiogenesis
Wnt
KDR
GJA1
TWIST1
ANGPT2
VEGFA
AXIN2
FLT1
BMP4

S17 Fig. Gene expression patterns of C-kit+CD45- cells based on heart chamber identity

C-kit+CD45- cells isolated from the four different heart chambers were included in an OPLS-DA model (n=32), predicting study participant identity. a) C-kit+CD45- cells isolated from right atrium tended to cluster separately as shown by the two first orthogonal principal components (PC) in a score plot. b-h) Gene expression patterns, demonstrated by orthogonal loading plots. C-kit+CD45- isolated from right atria expressed high levels of endothelial markers as well as markers involved in HIF, YAP/HIPPO, chemotactic, BMP and pro-angiogenic signaling. Genes have been color- and symbol-coded based on the corresponding gene annotation, as noted to the right of each figure. To improve visualization, some genes are included in more than one panel due to multiple annotations.
