# Supplementary material for: Human intracardiac SSEA4+CD34- cells show features of cycling, immature cardiomyocytes and are distinct from Side Population and C-kit+CD45- cells
Source: PLoS One. 2022 Jun 16;17(6):e0269985. doi: 10.1371/journal.pone.0269985 (PMC9202910; doi:10.1371/journal.pone.0269985)
Supplement: S1 Table — Abbreviations: NYHA, New York Heart Association; LVEF, left ventricular ejection fraction; ACEi, angiotensin-converting enzyme (ACE) inhibitors; ARB, angiotensin receptor blockers; ICD, implantable cardioverter defibrillator; CRT, cardiac resynchronization therapy; CRT-D, cardiac resynchronization therapy defibrillator; PCI, percutaneous coronary intervention; CABG, coronary artery bypass graft surgery. (PDF) [file pone.0269985.s018.pdf]

S1 Table. Clinical background of included heart failure patients.

Variable	Value
**Basic characteristics**	
Age, years (SD)	64 (10)
Sex, n	
Male	9
Female	0
Smoker, n	
Yes	0
No	3
Previous	6
Body weight, kg (SD)	83 (15)
BMI, kg/m^2 (SD)	26 (4.2)
**Cardiac function**	
Chronic Heart Failure, n (%)	9 (100)
Failing ventricles, n	
Left	3
Right	0
Both	6
Heart Failure etiology, n	
Idiopathic	4
Ischemia	3
Other	2
NYHA score at transplantation, n	
I	0
II	1
III	8
IV	0
LVEF before transplantation, % (SD)	30 (11)
**Therapy**	
ACEi/ARB	7
Beta Blockers	7
Ivabradine	1
Aldosterone Antagonists	6
Digoxin	0
Diuretics	8
Levosimendan	3
Antiplatelet	4
Anticoagulant	9
Nitrates	0
Antiarrhythmic drug	2
Statins	5
Pacemaker/ICD, n	
No	3
ICD	5
CRT	1
CRT-D	1
Mechanical circulatory support device (LVAD)	3

S1 Table, continued.

Variable	Value
**Comorbidites**	
Ischemic Heart Disease	3
Myocardial Infarction	3
Previous PCI	3
Previous CABG	0
Valvular disease	3
Arrhythmia	5
Peripheral Arterial Disease	0
Cerebrovascular Disease	2
Pulmonary Disease	1
Diabetes Mellitus	
No	3
Type 1	1
Type 2	5
Impaired Renal Function	6
Hypertension	1
Malignancy	0

NYHA, New York Heart Association; LVEF, left ventricular ejection fraction; ACEi, angiotensin-converting enzyme (ACE) inhibitors; ARB, angiotensin receptor blockers; ICD, implantable cardioverter defibrillator; CRT, cardiac resynchronization therapy; CRT-D, cardiac resynchronization therapy defibrillator; PCI, percutaneous coronary intervention; CABG, coronary artery bypass graft surgery
