# Supplementary material for: Human intracardiac SSEA4+CD34- cells show features of cycling, immature cardiomyocytes and are distinct from Side Population and C-kit+CD45- cells
Source: PLoS One. 2022 Jun 16;17(6):e0269985. doi: 10.1371/journal.pone.0269985 (PMC9202910; doi:10.1371/journal.pone.0269985)
Supplement: S2 Table — Descriptive statistics for each study participant. No organ donor control suffered from chronic heart failure. Abbreviations: CHF, chronic heart failure; IHD, ischemic heart disease; pMI, previous myocardial infarction; PAD, peripheral artery disease; CVD, Cerebrovascular Disease. (PDF) [file pone.0269985.s019.pdf]

S2 Table. Clinical background of included organ donors.

Donor	Age, years	Sex	Cause of death	Reason for heart being disqualified from transplantation	CHF	IHD	pMI	Arrhythmia	PAD	CVD	Pulmonary Disease	Diabetes Mellitus	Impaired Renal Function	Hyper-tension	Malignancy
1	62	M	Subarachnoid hemorrhage	Previous MAZE surgery	No	No	No	Atrial fibrillation, previous MAZE surgery	No	Subarachnoid hemorrhage	No	No	No	No	No
2	63	F	Ischemic brain edema after cardiac arrest	Ischemic Heart Disease	No	Yes	No	No	No	No	Emphysema	Type 2	Yes	Yes	No
3	52	M	Cardiac arrest, known substance addiction	Prolonged cardiac arrest, impaired function during echocardiogram in the acute setting	No	No	No	No	No	No	No	No	No	No	No
4	75	F	Cerebral hemorrhage	Age, previous myocardial infarction and atrial fibrillation	No	Yes	Yes	Atrial fibrillation	No	No	No	No	No	No	No

Descriptive statistics for each study participant. No organ donor control suffered from chronic heart failure. CHF, chronic heart failure; IHD, ischemic heart disease; pMI, previous myocardial infarction; PAD, peripheral artery disease; CVD, Cerebrovascular Disease
