# Supplementary material for: Human intracardiac SSEA4+CD34- cells show features of cycling, immature cardiomyocytes and are distinct from Side Population and C-kit+CD45- cells
Source: PLoS One. 2022 Jun 16;17(6):e0269985. doi: 10.1371/journal.pone.0269985 (PMC9202910; doi:10.1371/journal.pone.0269985)
Supplement: S3 Table — Antibodies used for flow cytometric analysis and sorting. Several fluorochrome conjugates were used for different staining panels. (PDF) [file pone.0269985.s020.pdf]

S3 Table. Antibodies used for flow cytometric analysis and sorting.

Antigen	Clone	Isotype	Clonality	Host	Company
CD34	8G12 (HPCA2)	IgG1	Monoclonal	Mouse	BD Biosciences
CD45	HI30 (RUO)	IgG1	Monoclonal	Mouse	BD Biosciences
CD144	16B1	IgG1	Monoclonal	Mouse	eBioscience
C-kit	104D2 (CE_IVD)	IgG1	Monoclonal	Mouse	BD Biosciences
CXCR4	12G5	IgG2a	Monoclonal	Mouse	Biolegend
SSEA3	eBioMC-631 (MC-631)	IgM	Monoclonal	Rat	eBioscience
SSEA4	eBioMC-813-70 (MC-813-70)	IgG3	Monoclonal	Mouse	eBioscience

Antibodies used for flow cytometric analysis and sorting. Several fluorochrome conjugates were used for different staining panels.
