# Supplementary material for: Human intracardiac SSEA4+CD34- cells show features of cycling, immature cardiomyocytes and are distinct from Side Population and C-kit+CD45- cells
Source: PLoS One. 2022 Jun 16;17(6):e0269985. doi: 10.1371/journal.pone.0269985 (PMC9202910; doi:10.1371/journal.pone.0269985)
Supplement: S4 Table — Based on a review of the previous literature, genes of interest (GOI) were selected for analysis. These included markers of cell type as well as pathways relevant for cardiac disease and stem-/progenitor cell biology. 94 assays—including the reference gene PPIA–were selected. (PDF) [file pone.0269985.s021.pdf]

S4 Table. Gene assays analyzed using BioMark and the 96x96 Dynamic Array™ IFC.

Gene	Cell type	Pathway	Comment
PPIA/CYPA			Reference gene
GJA1	Cardiomyocyte	WNT	
ACTC1	Cardiomyocyte		
GATA4	Cardiomyocyte		
GATA6	Cardiomyocyte		
MEF2A	Cardiomyocyte		
MEF2C	Cardiomyocyte		
MESP1	Cardiomyocyte		
NKX2.5	Cardiomyocyte		
TBX20	Cardiomyocyte		
TBX5	Cardiomyocyte		
TNNT2	Cardiomyocyte		
NOS3	Endothelial	HIF; BMP; VEGF	
ETS1	Endothelial	VEGF	
KDR	Endothelial	VEGF; Angiogenesis	
CD31	Endothelial		
CD34	Endothelial		
VWF	Endothelial		
NOTCH1	Endothelial, arterial	Notch	
EFNB2	Endothelial, arterial		
FLT4	Endothelial, lymphatic		
SOX18	Endothelial, lymphatic		
EPHB4	Endothelial, venous		
COL1A1	Fibroblast		
PDGFRA	Fibroblast		
PTPRC	Leucocyte		
ACTA2	Smooth muscle cell		
MYOCD	Smooth muscle cell		
SMTN	Smooth muscle cell		
PDGFRB	Smooth muscle cell; Pericyte		
APLNR	Stem cell	Chemotaxis	
ABCB1	Stem cell		
ABCG2	Stem cell		
ALDH1A1	Stem cell		
ALDH3A1	Stem cell		
ALPL	Stem cell		
BMI1	Stem cell		
ISL1	Stem cell		
KIT	Stem cell		
NES	Stem cell		
POU5F1	Stem cell		
SOX2	Stem cell		

S4 Table, continued.

Gene	Cell type	Pathway	Comment
TERT	Stem cell		
THY1	Stem cell		
WT1	Stem cell		
ADRB1		Adrenergic signalling	
FLT1		Angiogenesis	
IL10		Anti-inflammatory	
BMP2		BMP	
BMPR1A		BMP	
BMPR2		BMP	
ICAM1		BMP	
ID1		BMP	
MSX2		BMP	
BMP4		BMP; WNT	
CXCL12		Chemotaxis	
CXCR4		Chemotaxis	
FGF2		FGF	
FGFR1		FGF	
FGFR2		FGF	
EPAS1		HIF	
HIF1A		HIF	
NOS2		HIF	
EDN1		HIF; BMP	
TIMP1		HIF; Fibrosis	
ANGPT2		HIF; Fibrosis; Angiogenesis	
VEGFA		HIF; VEGF; Angiogenesis	
ALOX15		Lipid metabolism	
ALOX15B		Lipid metabolism	
NPPA		Natriuretic peptide	
NPPB		Natriuretic peptide	
NPR1		Natriuretic peptide	
HES1		Notch	
HEY1		Notch	
MYF5		Notch	
BAX		PI3k/Akt	
BCL2		PI3k/Akt; VEGF	
CCL2		Proinflammatory	
IL1B		Proinflammatory	
IL6		Proinflammatory	
AGTR1		Renin-angiotensin	
POSTN		TGF-beta	
SERPINE1		TGF-beta	
TGFB1		TGF-beta	
TGFBR1		TGF-beta	
CTGF		TGF-beta; YAP/HIPPO	

S4 Table, continued.

Gene	Cell type	Pathway	Comment
MYC		TGF-beta; YAP/HIPPO	
BIRC5		VEGF	
XIAP		VEGF	
AXIN2		WNT	
TWIST1		WNT	
CYR61		YAP/HIPPO	
STK4		YAP/HIPPO	
YAP1		YAP/HIPPO	

Based on a review of the previous literature, genes of interest (GOI) were selected for analysis. These included markers of cell type as well as pathways relevant for cardiac disease and stem-/progenitor cell biology. 94 assays - including the reference gene *PPIA* – were selected.
