# Supplementary material for: Human intracardiac SSEA4+CD34- cells show features of cycling, immature cardiomyocytes and are distinct from Side Population and C-kit+CD45- cells
Source: PLoS One. 2022 Jun 16;17(6):e0269985. doi: 10.1371/journal.pone.0269985 (PMC9202910; doi:10.1371/journal.pone.0269985)
Supplement: S5 Table — Based on a review of the previous literature, 9 cell cycle markers were chosen for analysis. (PDF) [file pone.0269985.s022.pdf]

S5 Table. Cell cycle regulators analyzed using standard qPCR.

Gene	Assay ID
AURKA	Hs01582072_m1
CCNA2	Hs00996788_m1
CCNB1	Hs01030099_m1
CDC20	Hs00426680_mH
CDC25B	Hs01582335_m1
CDK1	Hs00938777_m1
CDKN1A	Hs00355782_m1
CDKN1B	Hs00153277_m1
PCNA	Hs00696862_m1

Based on a review of the previous literature, 9 cell cycle markers were chosen for analysis.
