# Supplementary material for: Human intracardiac SSEA4+CD34- cells show features of cycling, immature cardiomyocytes and are distinct from Side Population and C-kit+CD45- cells
Source: PLoS One. 2022 Jun 16;17(6):e0269985. doi: 10.1371/journal.pone.0269985 (PMC9202910; doi:10.1371/journal.pone.0269985)
Supplement: S1 File — (DOCX) [file pone.0269985.s023.docx]

**Supporting methods and materials**

**Cell isolation procedure**

Biopsies were collected in cold phosphate buffered saline (PBS), washed with PBS to remove residual blood, weighed and cut into small pieces. Tissue pieces were digested with Liberase type TM 0.52 U/ml (Roche, Basel, Switzerland) and DNase type 1 2000 U/ml (Roche or Worthington Biochemical Corporation, Lakewood, NJ, USA) in DMEM:F12 (Thermo Fisher Scientific, Waltham, MA, USA) at 37°C for 4.5 hours under mild magnetic stirring. Cells were washed once, incubated for 10 minutes in 0.05% Tryspin-EDTA (Thermo Fisher Scientific) and resuspended in DMEM:F12 supplemented with 10% fetal bovine serum (FBS, Sigma-Aldrich St. Louis, MO, USA). The cells were filtered through a 250 μm metallic filter (Retsch GmbH, Haan, Germany) followed by 100 μm cell strainer filters (BD, Franklin Lakes, NJ, USA) to remove residual tissue fragments and cardiomyocytes.

Cells that would not undergo Side Population (SP) analysis were subjected to an epitope regeneration step, in order to optimize detection of protein markers, through incubation at 37°C for approximately 10-12 hours in DMEM:F12 supplemented with 5% FBS and 1mM EDTA (Sigma-Aldrich) under mild magnetic stirring. Afterwards, cells were centrifuged, resuspended in non-enzymatic cell dissociation solution (Sigma-Aldrich) and incubated for 10 minutes at 37°C.

Before processing cells for flow cytometric analysis, all cells were resuspended in erythrocyte lysis buffer (155 mM NH4Cl, 10 mM KHCO3 and 0.1 mM EDTA) and incubated for one minute at room temperature. Cells that would undergo SP analysis were then resuspended in SP staining medium (DMEM hepes, high glucose modified medium (Thermo Fisher Scientific) supplemented with 2% FBS) and filtered through 40 μm cell strainer filters (BD). All other cells were instead resuspended in cold FACS staining buffer (PBS supplemented with 5% FBS, 1% BSA (Sigma-Aldrich) and 2 mM EDTA) and filtered through 100 μm as well as 40 μm cell strainer filters.

**Staining for Side Population cells**

The cell concentration was adjusted to 10^6^ cells/ml in staining medium. 5 μM Fumitremorgin C (FTC, Sigma-Aldrich) and 100 μM Verapamil (Sigma-Aldrich) were included as specific efflux protein inhibitors in separate negative control samples. Additionally, 15 mM sodium azide (Sigma-Aldrich) diluted in 50 mM 2-Deoxy-D-glucose (Sigma-Aldrich, dissolved in staining medium) was used as a general inhibitor of metabolism in a separate negative control sample. The inhibitor concentrations were in line with previous experimental protocols used to isolate SP cells [1]. When SP inhibitors were used, samples were pre-incubated with the inhibitors for 20 min, and inhibitors were added in all subsequent steps until being kept on ice. Hoechst 33342 (Thermo Fisher Scientific) was added at a concentration of 5 μg/ml followed by incubation for 45 min at 37°C. Cells were then washed with staining medium and incubated without Hoechst during 45 min at 37°C. Finally, cells were washed with 4°C FACS staining buffer. From this step, and until FACS sorting, the cells were kept strictly on ice and centrifuged in a chilled centrifuge in order to avoid further Hoechst efflux.

**Antibody staining and fixation**

The cell concentration was adjusted to 1-2 10^6^ cells/ml in staining medium followed by incubation with 7-AAD (Thermo Fisher Scientific, for dead cell discrimination) and antibodies at 4°C for 30 min (S3 Table). Cold FACS staining buffer was then added, and cells filtered through a 40 μm cell strainer filter. Finally, cells were washed with FACS staining buffer and stored on ice in darkness until analysis.

Due to practical reasons, samples were in some cases subjected to prolonged storage on ice. To protect mRNA from storage induced degradation, samples were in these cases fixated after antibody incubation. For some experiments where material was abundant and analysis could be performed with a minimal time delay, both fixated and not fixated samples were included and sorted, to enable statistical correction for any biases specifically related to fixation.

After filtering, cells subjected to fixation were washed with cold RNase free PBS (Thermo Fisher Scientific), followed by fixation with 4% formaldehyde. For samples which had antibody panels that enabled staining with DAPI (Thermo Fisher Scientific), this was also added (28 μM) during fixation in order to improve the identification of cells. Cells were incubated at 4°C under darkness for 20 min, followed by two washes with cold RNase free PBS.

**Flow cytometric analysis and sorting**

FACS analysis was carried out on a FACSaria II cell sorter (BD). Appropriate lasers and filters were used for all fluorochromes included. SP was analyzed using a 375 nm near UV laser, using a 450/20 BP filter for Hoechst blue and a 670 LP filter for Hoechst red, respectively. Data analysis was conducted using FACSdiva version 6.1.1 (BD). An overview of the gating strategy is provided in S2 Fig. Cells were identified based on DAPI staining (for fixed cells, as non-fixed cells were not permeable for the DAPI staining) as well as forward scatter (FSC) versus side scatter (SSC). Dead cells were excluded based on 7‑AAD staining. Background antibody staining was determined by appropriate isotypic controls. For SSEA4 and C‑kit, a gating strategy to minimize the risk of false positive cells was used (isotypic control in the range of approximately 0.001–0.02%). Gates for CD34 and CD45 were on the other hand set to get as good discrimination between the SSEA4+CD34‑/SSEA4+CD34+, C‑kit+CD45‑/C‑kit+CD45+ and SP CD45‑/SP CD45+ populations as possible. For all other antibodies, approximately 0-2% false positive cells were included, based on isotypic controls. For SP, gating was conducted using the most effective inhibitor (verapamil, and sometimes sodium azide/2-Deoxy-D-glucose) to set a morphological SP gate. Isotypic and inhibitor controls were subtracted when statistics were calculated. For cell sorting to qPCR, cells were sorted into 1.5-ml tubes with either RLT buffer including DTT (Qiagen, Hilden, Germany) or RNase free PBS (Thermo Fisher Scientific) for non-fixed and fixed cells, respectively. Cells were stored in a freezer at –80 ° C until further analysis.

**RNA isolation, preparation, cDNA synthesis and preamplification**

Kits and equipment from Qiagen were used to isolate total RNA. Briefly, protocols of the RNeasy Plus/Mini and Plus/Micro Kits were applied as appropriate, depending on the number of cells per sample. Residual genomic DNA was removed using either gDNA columns or DNase1 treatment. The FFPE kit was used for formaldehyde-fixated cells. Reverse transcription of mRNA to cDNA was performed using the GrandScript cDNA Synthesis Kit (TATAA Biocenter, Gothenburg, Sweden).

RNA concentration was measured using digital droplet PCR (Bio-Rad Laboratories Hercules, CA, USA). First, a standard curve was established based on RNA extracted from whole human cardiac tissue. Original RNA concentration was measured using Nanodrop 2000 (Thermo Fisher Scientific), after which the RNA underwent serial dilution from 50 to 0.00005 pg/µl. RNA quantification was analyzed using TaqMan probe assays (Thermo Fisher Scientific) in a multiplex reaction. Two reference genes were used, 18S VIC-MGB Hs03003631_g1 and PPIA FAM-MGB Hs99999904_m1, to enable a wide measurement interval. Triplicates from each dilution were included with 2x ddPCR supermix for probes (no dUTP, Bio-Rad). The reaction mixes were converted into droplets in the QX200 AutoDG Droplet Digital PCR System. The thermal cycling was carried out on the T100 Thermal Cycler. Droplets were analyzed with the QX200 Droplet Reader. The average concentrations (copies/µl) of *PPIA* and *18S* were fitted against the theoretical total RNA concentrations using regression analysis, resulting in two mathematical functions. To estimate the total RNA concentration for each study sample, 2 µl of cDNA from each sample were analyzed for *PPIA* and *18S* by ddPCR. The *PPIA* and *18S* concentrations were entered into the mathematical functions. The resulting estimated total RNA concentrations were then used to standardize sample amounts for pre-amplification.

Due to low cDNA amount, all samples underwent preamplification. For the BioMark arrays, 5 µl (maximum of 0.3 ng) cDNA was preamplified using an assay mix (diluted 1:5) for 22 cycles with PreAmp GrandMaster Mix (TATAA Biocenter). When analyzing cell cycle regulators, a separate preamplification of 5 µl cDNA was performed using a separate assay mix (diluted 1:5) at 14 cycles with the TaqMan preAmplification Master Mix (Thermo Fisher Scientific).

**Gene expression analysis**

Based on a review of the previous literature, 93 genes of interest were selected for analysis. These included markers of cell type as well as pathways relevant for cardiac disease and stem-/progenitor cell biology. 94 assays (S4 Table), including the reference gene *PPIA*, were selected (Thermo Fisher Scientific). qPCR was performed using the BioMark (Fluidigm, San Francisco, CA, USA) and the 96x96 Dynamic Array™ IFC (Integrated Fluidic Circuit) at TATAA Biocenter. ValidPrime™ assays (TATAA Biocenter) were included in order to correct for possible gDNA amplification. qPCR was performed using TATAA Probe GrandMaster® Mix Low ROX (TATAA Biocenter) and the GE 96.96 Dynamic Array™ Sample & Assay Loading Reagent Kit (P/N 85000802-R, Fluidigm). No template controls (NTCs) for the qPCR were included in the sample plates. All samples were analyzed in single reactions. Data was analyzed using the Fluidigm Real-Time PCR Analysis Software using the linear derivative baseline correction. No amplification in the qPCR-NTC was detected. ValidPrime correction was done using the GenEx software (MultiD Analyses, Gothenburg, Sweden).

Cell cycle regulators were analyzed using standard reagents and instruments from Applied Biosystems (Thermo Fisher Scientific). The TaqMan™ Gene Expression Master Mix, TaqMan gene expression assays (S5 Table) and the ABI7900HT instrument were used.

**Statistical analysis**

Groupwise comparisons on flow cytometry statistics were carried out using analysis of variance (ANOVA) with Post-Hoc Tukey’s test, using the R packages car and emmeans, respectively. For comparisons where a block-variable could be used (i.e. comparisons between different locations and efflux inhibitors), a mixed model was fitted with study subject as random factor, using the lme4 R package. For non-normal distributed data, log transformation was performed. If the distribution was still determined to be non-normal, or if variance was determined to be non-equal, a non-parametric test was carried out using Prentice test in the muStat R package. Data distribution and equal variance were assessed visually. Non-parametric pairwise comparisons between different groups were carried out using Wilcoxon rank sum test. Paired Wilcoxon rank sum test was used when applicable (i.e. when comparing different locations or efflux inhibitors). P-values for non-parametric comparisons were adjusted for multiple testing using the Hochberg method as implemented in the p.adjust method in R.

To perform differential gene expression analysis, CT values were first imported into R. From the initial dataset, 24 samples were removed due to low quality and twelve due to high fraction of UD values (>80 %). Quality criteria for exclusion were low cell count and deviating cell count in relationship to RNA concentration. Five genes were also removed due to high UD fraction (*ISL1, ALDH3A1, MESP1, MYF5, TERT*). UD values were then imputed per gene as the maximum CT value for each gene + 2. CT values were normalized against PPIA by subtracting the reference gene CT with genes of interest. Next, delta CT (dCT) values were corrected for fixation using a linear model, where the corrected dCT was modeled by the fixated dCT. To fit the model, all paired samples were used where non-UD values were available for both fixated and non-fixated samples from the same heart and chamber. Fitted model coefficients were then used to adjust fixated dCT in order to subtract away the effect of fixation. Two genes (*AUTKB and MKi67*) were removed in this step because they could not be adjusted. Next, mean dCT was calculated across technical replicates while disregarding any UD values. Following this, dCT was also corrected for heart failure status (present/absent) using ComBat [2], and mean dCT was calculated for each heart (across samples from different chambers). These mean dCT values were subsequently used in multivariate analyses as well as to identify differentially expressed genes between cell populations of interest. For analyses that specifically explored differences between failing / non-failing hearts, data was only corrected for fixation. Similarly, for analyses exploring differences between the four chambers of the heart, the mean calculations step was omitted. The analysis was performed with the miodin package [3], which relies on limma [4] for the statistical analysis. P-values for differentially expressed genes were adjusted for multiple testing with Benjamini Hochberg correction. Genes with log_2_ fold change > 1 and false discovery rate < 5 % were considered statistically significant.

Unsupervised Principal Component Analysis (PCA) was performed to determine clustering between SSEA4+CD34‑, SP CD45‑, SP CD45+ and SSEA4+CD34‑ cells in score plots. The corresponding loading plots showed the underlying correlation structure of the 87 genes analyzed. PCA is an unbiased statistical method of summarizing systematic variation in multivariate data through a few so-called principal components (PCs). By visualizing the PC-scores for individual observations in a dot-plot (“score plot”), clustering of observations can be identified, implying a systematic grouping of observations based on the underlying data variables. The corresponding weights of the data variables can also be visualized through dot-plots (“loading plots”). The variables responsible for the systematic clustering of observations can thus be identified.

Orthogonal Projections to Latent Structures Discriminant Analysis (OPLS-DA) models for the prediction of cell population identity were also calculated to determine the regression coefficients of the respective genes. OPLS-DA is a method of constructing a predictive model of a categorical variable, based on previously collected multivariate data. OPLS-DA can thus be used as an alternative to PCA in identifying variables that predict categorical group identity, such as cell population identity. Regression coefficients can also be calculated for the individual variables, to show the predictive direction of the different variables. Variables with coefficients >0 are positively associated with the indicated group, whereas variables with coefficients <0 are negatively associated. Error bars may be displayed for coefficients at the 95% confidence level, estimated by a jack-knifing procedure. Coefficients where the error bars did not cross 0 are considered most reliable and significant. Cross-validation was performed using 7 randomly constructed groups. Outliers were analyzed based on Hotellings T2 and DmodX, as well as t1/u1 plots for the OPLS models. No outliers were identified. *CD34*, *PTPRC* (CD45) and *KIT* (C‑kit) were included in the FACS sorting algorithms. In order to avoid statistical bias when analyzing the clustering of cell populations as well as the resulting regression coefficients, *CD34*, *PTPRC* and *KIT* were not included in the PCA/OPLS-DA models. When included, the models however yielded similar results (data not shown).

Secondly, in order to compare the cell populations to a non-cardiomyocyte reference population, differentially expressed genes as compared to the MP were determined for SSEA4+CD34‑, SP CD45‑, SP CD45+ and C-kit+CD45- cells respectively. Significantly differentially expressed genes were visualized in heatmaps. The heatmap color scale was centered with a mean of 0 and a standard deviation of 1, for each gene. Hierarchical clustering analysis with complete linkage was calculated using hclust function in R, based on all differentially expressed genes. As only differentially expressed genes were included, an overall clustering of samples based on cell population identity was to be expected. *CD34*, *PTPRC* and *KIT* were therefore included in the heatmap calculations. These markers were used as positive controls in validating the FACS sorting algorithm.

To analyze the differences in gene expression patterns between failing and organ donor hearts for SSEA4+CD34‑ cells, an OPLS-DA model was calculated to determine the presence of heart failure. Due to the limited number of data points (donors = 3, heart failure = 9), the leave-one-out strategy for cross-validation was applied using 12 cross-validation groups. No outliers were identified. As only the SSEA4+CD34‑ population was included, *CD34*, *PTPRC* and *KIT* could be included in the model.

To analyze the differences in gene expression patterns between different chambers of the heart for each respective cell population, the individual data points for each heart chamber were used instead of a mean value for all heart chambers. In order to correct for the inter-individual variation, an OPLS-DA model was calculated to determine the study participant identity. The orthogonal score and loading vectors were then analyzed to determine clustering of samples and genes – similar to a PCA. Cross-validation was performed using 7 randomly constructed groups. A maximum of 1 outlier per model was excluded. As the comparisons were performed within each population, *CD34*, *PTPRC* and *KIT* could be included in the models.

To analyze the differences in cell cycle regulators between the different cell populations, data quality control and correction was conducted as described previously for the 94 gene qPCR data analysis. Instead of imputing values for UD values however, UD values were instead assigned a value of 0. A mean dCT value was calculated for each heart, based on the available samples from the different heart chambers. Prior to PCA model fitting, dCT values were transformed by adding 1 to each dCT value, followed by log transformation.

All statistical analyses were performed using Simca v. 15 (Sartorius Stedim Data Analytics AB, Umeå, Sweden) or R v. 4.0.2 (R Core Team 2020)[5].

**References**

1. Sandstedt J, Jonsson M, Kajic K, Sandstedt M, Lindahl A, Dellgren G, et al. Left atrium of the human adult heart contains a population of side population cells. Basic Res Cardiol. 2012;107(2):1-10. doi:10.1007/s00395-012-0255-7

2. Leek JT, Johnson WE, Parker HS, Jaffe AE, Storey JD. The sva package for removing batch effects and other unwanted variation in high-throughput experiments. Bioinformatics. 2012;28(6):882-3. doi:10.1093/bioinformatics/bts034

3. Ulfenborg B. Vertical and horizontal integration of multi-omics data with miodin. BMC Bioinformatics. 2019;20(1):649. doi:10.1186/s12859-019-3224-4

4. Ritchie ME, Phipson B, Wu D, Hu Y, Law CW, Shi W, et al. limma powers differential expression analyses for RNA-sequencing and microarray studies. Nucleic Acids Res. 2015;43(7):e47. doi:10.1093/nar/gkv007

5. Team RC. R: A Language and Environment for Statistical Computing. R Foundation for Statistical Computing. 2020.
