# Supplementary material for: Human intracardiac SSEA4+CD34- cells show features of cycling, immature cardiomyocytes and are distinct from Side Population and C-kit+CD45- cells
Source: PLoS One. 2022 Jun 16;17(6):e0269985. doi: 10.1371/journal.pone.0269985 (PMC9202910; doi:10.1371/journal.pone.0269985)
Supplement: S12 Fig — To determine differentially expressed genes by C-kit+CD45- cells, MP was used as reference population (n = 10). Significantly differentially expressed genes at an FDR of < 5% are included in the heatmap. C-kit+CD45- cells expressed higher levels of IL10, CXCR4 and ADRB1 (c). Markers involved in TGFβ and BMP signaling were on the other hand expressed at lower levels (d). The heat color scale has been centered with a mean of 0 and a standard deviation of 1, for each gene. Hierarchical clustering resulted in separation between C-kit+CD45- and MP cells. Genes and populations have been color-coded based on the corresponding annotations, as noted to the right of each figure. To improve visualization, some genes are included in more than one panel due to multiple annotations. HF = Heart failure patient, Don = Donor. (PDF) [file pone.0269985.s012.pdf]

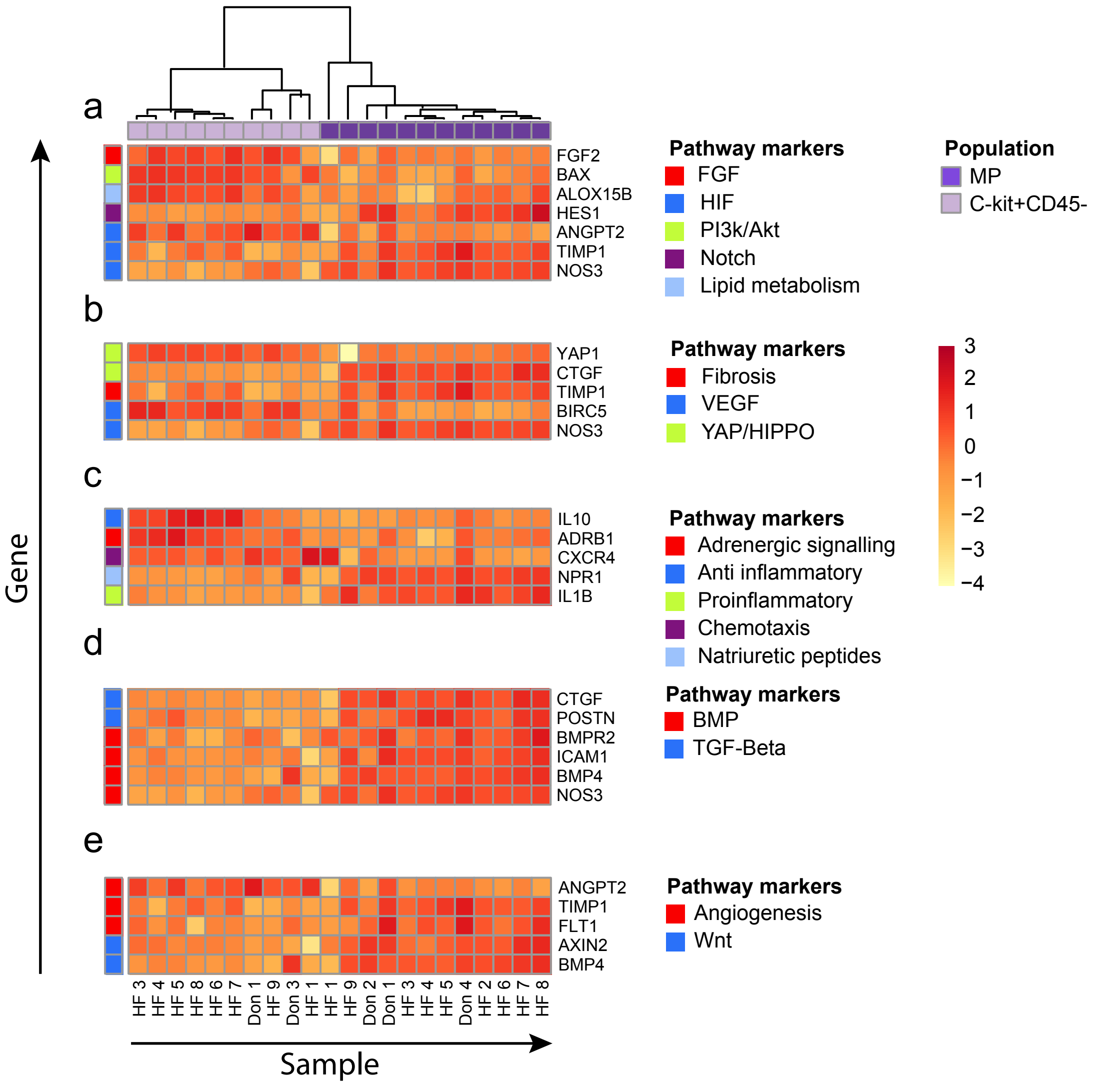

S12 Fig. Differentially expressed pathway marker genes by C-kit+CD45- cells

To determine differentially expressed genes by C-kit+CD45- cells, MP was used as reference population (n=10). Significantly differentially expressed genes at an FDR of < 5% are included in the heatmap. C-kit+CD45- cells expressed higher levels of *IL10*, *CXCR4* and *ADRB1* (c). Markers involved in TGFβ and BMP signaling were on the other hand expressed at lower levels (d). The heat color scale has been centered with a mean of 0 and a standard deviation of 1, for each gene. Hierarchical clustering resulted in separation between C-kit+CD45- and MP cells. Genes and populations have been color-coded based on the corresponding annotations, as noted to the right of each figure. To improve visualization, some genes are included in more than one panel due to multiple annotations. HF = Heart failure patient, Don = Donor.
